# Patient-derived organoids identify tailored therapeutic options and determinants of plasticity in sarcomatoid urothelial bladder cancer

**DOI:** 10.1038/s41698-023-00466-w

**Published:** 2023-11-02

**Authors:** Michele Garioni, Viviane J. Tschan, Lauriane Blukacz, Sandro Nuciforo, Romuald Parmentier, Luca Roma, Mairene Coto-Llerena, Heike Pueschel, Salvatore Piscuoglio, Tatjana Vlajnic, Frank Stenner, Hans-Helge Seifert, Cyrill A. Rentsch, Lukas Bubendorf, Clémentine Le Magnen

**Affiliations:** 1 Institute of Medical Genetics and Pathology, University Hospital Basel, University of Basel, Basel, Switzerland; 2 Department of Urology, University Hospital Basel, Basel, Switzerland; 3 Department of Biomedicine, University Hospital Basel, University of Basel, Basel, Switzerland; 4 Division of Medical Oncology, University Hospital Basel, Basel, Switzerland

**Keywords:** Bladder cancer, Cancer models

## Abstract

Sarcomatoid Urothelial Bladder Cancer (SARC) is a rare and aggressive histological subtype of bladder cancer for which therapeutic options are limited and experimental models are lacking. Here, we report the establishment of a long-term 3D organoid-like model derived from a SARC patient (*SarBC-01*). SarBC-01 emulates aggressive morphological, phenotypical, and transcriptional features of SARC and harbors somatic mutations in genes frequently altered in sarcomatoid tumors such as *TP53* (p53) and *RB1* (pRB). High-throughput drug screening, using a library comprising 1567 compounds in SarBC-01 and conventional urothelial carcinoma (UroCa) organoids, identified drug candidates active against SARC cells exclusively, or UroCa cells exclusively, or both. Among those, standard-of-care chemotherapeutic drugs inhibited both SARC and UroCa cells, while a subset of targeted drugs was specifically effective in SARC cells, including agents targeting the Glucocorticoid Receptor (GR) pathway. In two independent patient cohorts and in organoid models, GR and its encoding gene *NR3C1* were found to be significantly more expressed in SARC as compared to UroCa, suggesting that high GR expression is a hallmark of SARC tumors. Further, glucocorticoid treatment impaired the mesenchymal morphology, abrogated the invasive ability of SARC cells, and led to transcriptomic changes associated with reversion of epithelial-to-mesenchymal transition, at single-cell level. Altogether, our study highlights the power of organoids for precision oncology and for providing key insights into factors driving rare tumor entities.

## Introduction

While more than 90% of bladder cancers present as “conventional” urothelial carcinomas (UroCa), additional histological subtypes have been described which are often clinically aggressive and challenging to treat^1^. Among those subtypes, sarcomatoid urothelial bladder cancer (SARC) represents <1% of all bladder cancers, typically associates with early metastatic spread to distant organs, and is linked to poor prognosis^2–5^. At the histological level, SARC is characterized by tumor areas that are indistinguishable from sarcoma and frequently present with admixed conventional UroCa components^6,7^. Morphological features of SARC vary from nondescript spindle cells to undifferentiated pleomorphic patterns, sometimes with heterologous components such as osteosarcoma or angiosarcoma. As SARC is often found concomitant to conventional UroCa, it has been proposed that both components may share a common ancestor^4^. Supporting this hypothesis, recent sequencing efforts have suggested that SARC exhibit genomic features common with UroCa and may evolve via the dysregulation of epithelial-to-mesenchymal transition (EMT) pathways^4,8^. Yet SARC tumors differ from conventional UroCa by an enrichment of *TP53*, *RB1* and *PIK3CA* mutations and an enhanced expression of genes linked to chromatin remodelling and EMT^4^. Despite these specific features, it is recommended to treat patients with mixed histology the same way as patients with pure UroCa, yet a consensus for the correct clinical management of patients with predominant variant histology is still lacking^9^.

**Fig. 1:**
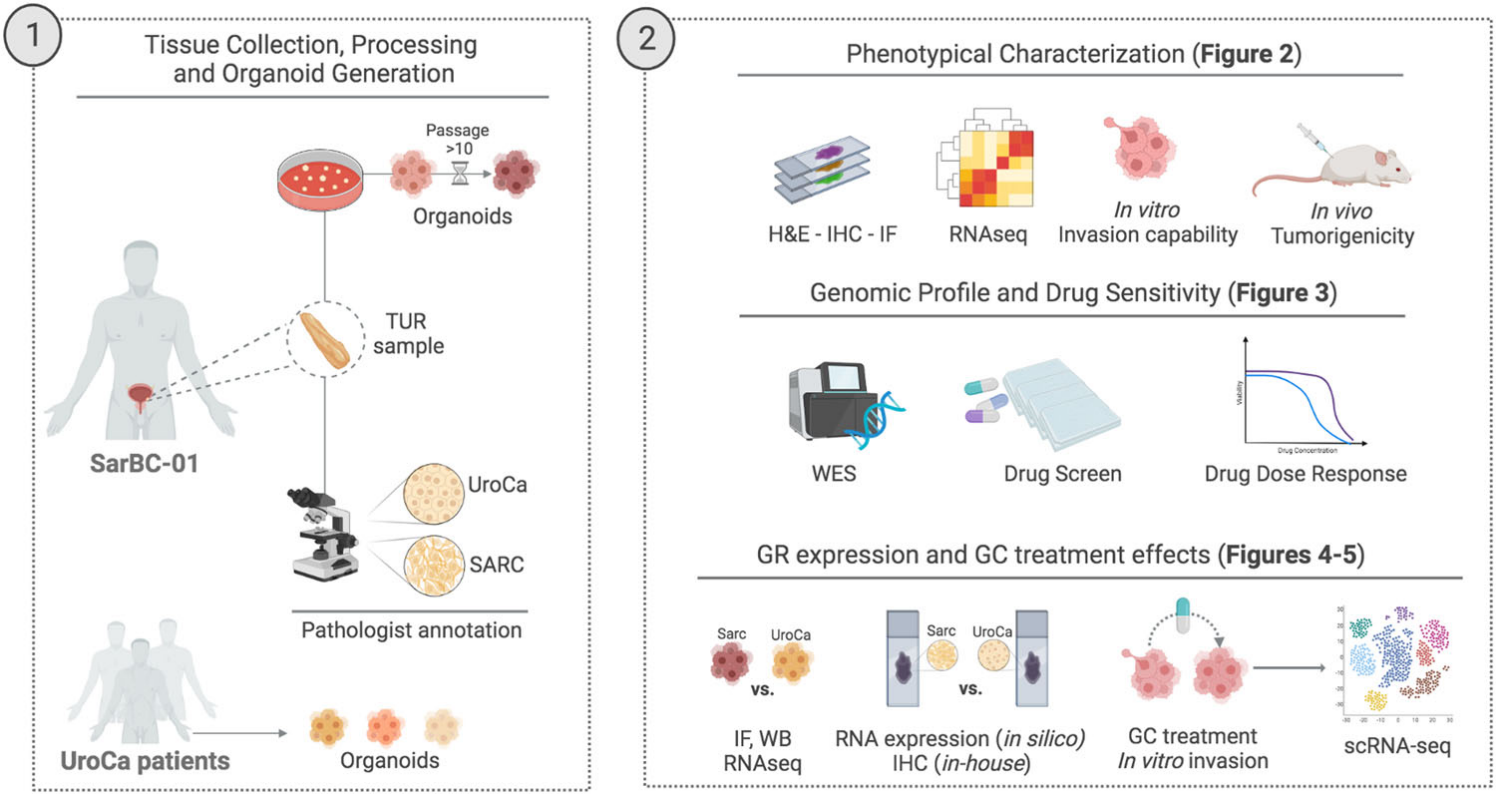
Cartoon depicting the distinct morphological components of the patients’ tumor and the experimental workflow used to generate organoids (1) and subsequent analyses (2). UroCa urothelial carcinoma, SARC sarcomatoid urothelial bladder cancer, TUR transurethral resection, H&E hematoxylin and eosin, IHC immunohistochemistry, IF immunofluorescence, WES whole exome sequencing, WB western blot, GR glucocorticoid receptor, GC glucocorticoids. Created with BioRender.com.

The recent advent of 3D in vitro models such as patient-derived organoids (PDOs) has opened new avenues to decipher the pathogenesis and therapeutic vulnerabilities of a wide range of tumors, including bladder cancer^10,11^. A lack of experimental models emulating rare entities, such as SARC, however hampers the efforts to decipher mechanisms driving such diseases and the development of tailored clinical strategies. Here, we established a long-term in vitro PDO model from a patient harboring SARC (***SarBC-01***) and primarily aimed at characterizing its phenotypic, molecular, and functional features to demonstrate its relevance for modelling SARC (see experimental flow in Fig. 1). Secondarily, we aimed at exploiting SarBC-01 organoids to identify putative therapeutic options for SARC and factors that may be associated with BC progression.

## Results

### Generation of a long-term organoid model derived from a SARC patient, which retains phenotypic characteristics of SARC

**Fig. 2:**
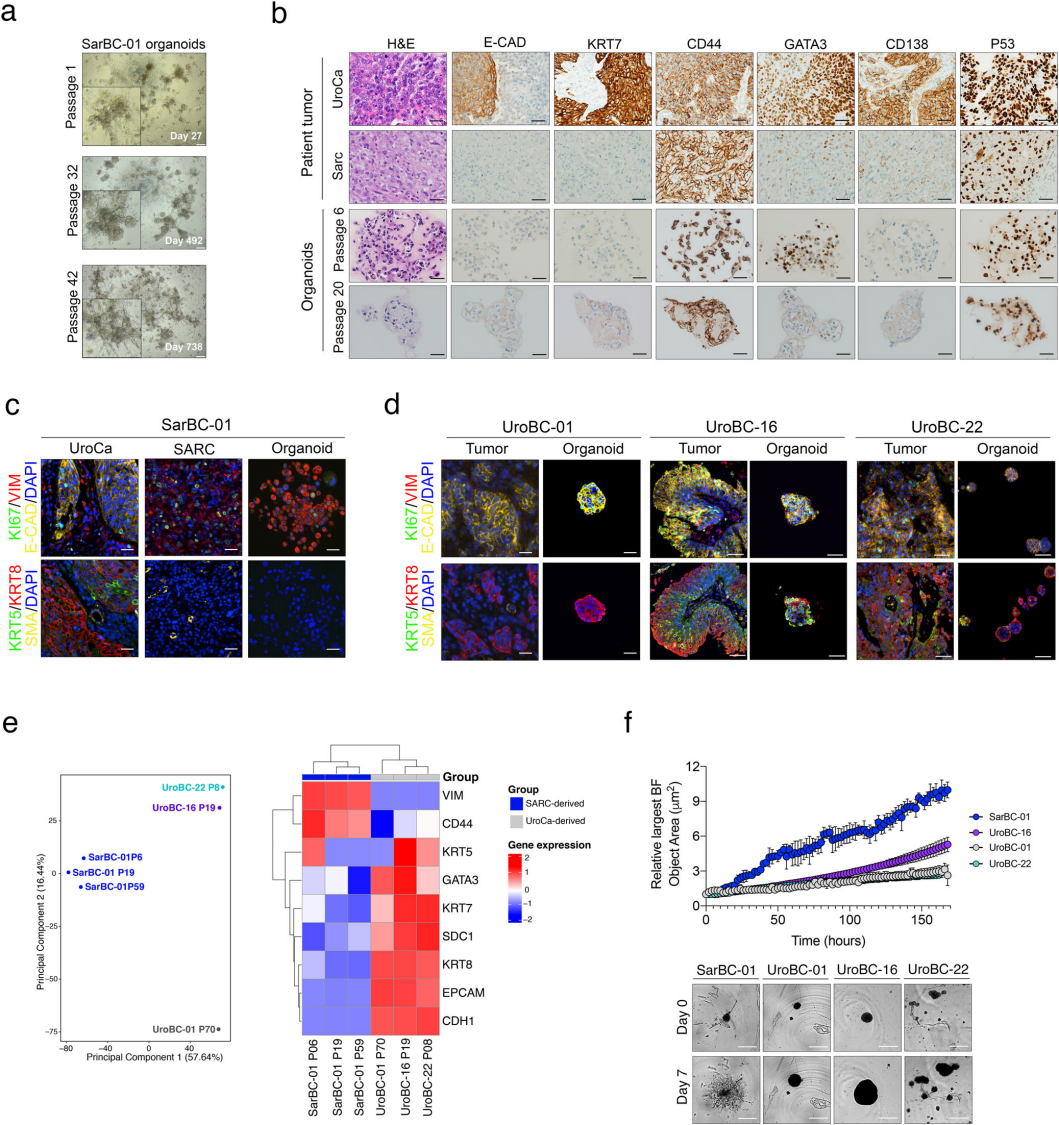
Generation of a long-term in vitro model derived from a SARC patient, which displays mesenchymal and invasive properties. **a** Representative bright field images of SarBC-01 organoids at passage 1 (27 days of culture), passage 32 (492 days of culture), and passage 42 (728 days of culture). Scale bars represent 200 μm. **b** Phenotypic analyses of the distinct tumor components (UroCa, SARC) and derived organoids at early and late passage (passage 6 and 20, respectively). Shown are representative H&E images and IHC staining for the indicated antibodies. Scale bars represent 50 μm. **c**, **d** Immunofluorescence analyses of SarBC-01 (**c**) and UroBC-01, UroBC-16, and UroBC-22 (**d**) tumor and organoids pairs. Shown are representative images for the indicated antibodies. *Vim: Vimentin, E-cad: E-cadherin, SMA: Smooth Muscle Actin*. DAPI: 4’,6-diamidino-2-phenylindole. Scale bars represent 50 μm. **e** Left panel: Principal Component Analysis (PCA) based on comparison of RNA-sequencing gene expression profiles of SarBC-01 organoids (passage 6, passage 19, passage 59), UroBC-01 (passage 70), UroBC-16 (passage 19), and UroBC-22 (passage 8). Right panel: Heatmap depicting unsupervised clustering analysis based on relative expression of selected epithelial- and mesenchymal-associated genes. **f** Top panel: Largest bright field object area measured over time for spheroids generated from SarBC-01 cells and UroBC-01 cells Analyses were performed using a mixed-effects analysis (*P* = 0.0001). Data are represented as means of three spheroids (relative to day 0 for each) and the error bars represent standard deviations (SD). Two independent biological replicates were performed and one representative replicate is shown. Bottom panel: representative images of SarBC-01 (passage 40), UroBC-01 (passage 39), UroBC-16 (passage 16), and UroBC-22 (passage 9) spheroids at day 0 and day 7 are shown. Scale bars represent 800 µm.

We generated organoid cultures out of a fragment of a tumor derived from a SARC patient (***SarBC-01***), adapting a previously published protocol^10^. SarBC-01 organoids, were maintained in culture at long-term (> 75 passages and >3 years in culture) and underwent multiple cycles of freezing and thawing, without notable difference of growth in culture (Fig. 2a). The original patient tumor displayed a wide spectrum of differentiation patterns ranging from poorly differentiated UroCa to SARC components, the latter being characterized by malignant cells with a spindle-like morphology (Fig. 2b). The UroCa component retained expression of epithelial markers such as E-cadherin and Cytokeratin (KRT) 7, and expressed high levels of CD138 and GATA3. In contrast, these proteins were absent or expressed at very low level in the SARC counterpart, while CD44 and p53 were highly expressed in both components. Notably, “early” and “late” passage organoids (passage 6 and 20, respectively) displayed marker expression patterns consistent with a SARC-like phenotype, suggesting that they may derive from the SARC component or that this tumor component may have preferentially grown in culture (Fig. 2b). Similar to the patients’ SARC tumor component and in contrast to the UroCa component, organoids were negative for additional epithelial markers (KRT5, KRT8), yet showed a strong expression of Vimentin, hallmark of mesenchymal-like cells (Fig. 2c).

### SarBC-01 organoids display aggressive mesenchymal features

To provide comparative models of UroCa for further analyses, we used organoid models derived from eight patients with conventional UroCa that were newly established in our laboratory (***UroBC-01, UroBC-02, UroBC-06, UroBC-015, UroBC-16, UroBC-18, UroBC-19, UroBC-22***). In stark contrast with SarBC-01 cells, UroCa organoids expressed high levels of the epithelial marker E-cadherin, and lacked expression of Vimentin, consistent with an UroCa phenotype (Fig. 2d and Supplementary Fig. 1A). Noteworthily, most of the UroCa organoid models displayed a pure KRT8^+^KRT5^–^ luminal-like phenotype with the exception of UroBC-16 and UroBC-22, exhibiting a mixed phenotype comprising KRT5^+^ and KRT8^–^ cells. Five out of eight UroCa models were not maintained over several passages and were therefore exclusively used for phenotypic analyses. In contrast, UroBC-01, UroBC-16, and UroBC-22 were stably maintained at long-term (i.e., more than 10 passages), thereby allowing their use in omics and functional analyses.

To understand the molecular basis underlying their distinct phenotype, we performed bulk mRNA sequencing analysis in SarBC-01 organoids at three distinct passages (passage (P)6, P19, P59) and in UroBC-01 (P70), UroBC-16 (P19), and UroBC-22 (P8). Following principal component analysis, SarBC-01 organoids clustered together and separately from the UroCa models (Fig. 2e, left panel). In addition, consistent with their close phenotypic features, UroBC-16 and UroBC-22 clustered together separately from UroBC-01. Comparing expression profiles of a panel of genes associated with stromal and epithelial cells, unsupervised hierarchical clustering discriminated SARC-derived organoids (SarBC-01 P6, P19, P59) and UroCa-derived models (UroBC-01, UroBC-16, UroBC-22; Fig. 2e, right panel). In particular, SarBC-01 organoids distinguished themselves by high expression of Vimentin and CD44 and low expression of epithelial-like markers (*GATA3, KRT7, SDC1, KRT8, EPCAM, CDH1*). With the exception of KRT5 which was more expressed in UroBC-16 and UroBC-22, the three UroCa models displayed comparable levels of urothelial-associated markers.

Despite an estimated longer doubling-time (Supplementary Fig. 1b), SarBC-01 displayed a significantly higher invasive capacity in vitro as compared to UroBC-01, UroBC-16, and UroBC-22, consistent with a more aggressive phenotype (mixed-effects analysis, *P* = 0.0001; Fig. 2f and Supplementary Videos 1–4). These in vitro invasive features correlated with a faster tumorigenic capacity in vivo in the SarBC-01 model as compared to UroBC-01 (2/2 xenografts generated for each model, average time between palpable tumor and end-point of 35 days in SarBC-01 vs. 210 days in UroBC-01; Supplementary Fig. 2). Xenografts generated out of SarBC-01 cells displayed rhabdoid features and high expression of Vimentin, while UroBC-01-derived xenografts were negative for this marker (cells with human origin; Supplementary Fig. 2). Notably, portions of xenografts derived from SarBC-01 cells displayed intermediate phenotypes with poorly differentiated UroCa-like morphology and partial recovery of expression of epithelial markers, reflecting the spectrum of heterogeneity found in the patient sample and suggesting that SarBC-01 cells may be prone to phenotypic changes in an in vivo environment (Supplementary Fig. 2).

### SarBC-01 organoids harbor genomic alterations which are shared with their parental tumor and are enriched in sarcomatoid malignancies

**Fig. 3:**
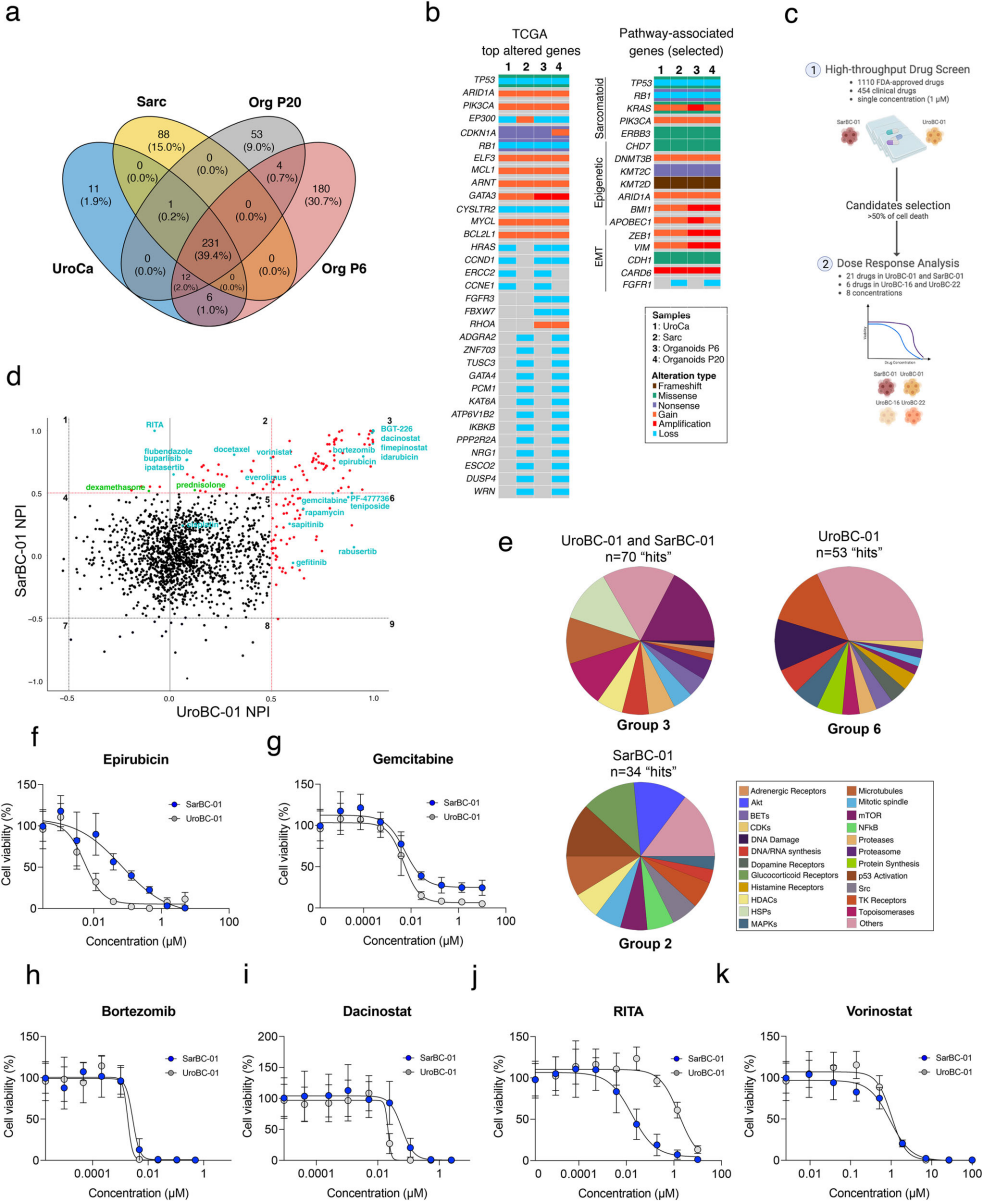
Identification of genomic drivers and drug sensitivities in SarBC-01 organoids. **a** Venn diagram depicting the number of shared mutations among SarBC-01 patient tumors’ components and derived organoids at early and late passages detected using whole exome sequencing (WES). Numbers in brackets indicate the proportion of shared mutations in each group. **b** Oncoplots depicting genomic alterations in the distinct SarBC-01 associated samples, as revealed by whole exome sequencing. Shown are alterations among the top 100 genes commonly mutated in BC (TCGA, left panel) and among selected genes associated with sarcomatoid cancers, epigenetic, and EMT pathways (right panel). Only alterations found in at least two samples are represented. A complete list of genomic alterations can be found in Supplementary Data 1. **c** Schematic representation of the workflow used for the high-throughput drug screening and subsequent dose response analyses. **d** Dot plot depicting normalized percentage inhibition (NPI) measured for SarBC-01 and UroBC-01, each dot representing one drug. “Hits” are defined as compounds associated with >50% inhibition as compared to negative controls (red dots, above the red line threshold). Compounds with inhibitory effects (“hits”) in UroBC-01 only (top right, group 6), SarBC-01 only (bottom left, group 2), and in both lines (top left, group 3) were identified. Drugs that have been further tested in dose response analyses are labelled in cyan. Labelled in green are the glucocorticoids dexamethasone and prednisolone. **e** Pie charts summarizing the mode of action categories of drugs identified as hits for both UroBC-01 and SarBC-01 (top left, group 3), UroBC-01 only (top right, group 6), and SarBC-01 only (bottom left, group 2). **f**–**k** Dose-responses curves for SarBC-01 and UroBC-01 organoids treated with a selected panel of drugs. Examples include standard of care compounds (**f**, **g**), drugs identified as “hit” in both SarBC-01 and UroBC-01 lines (**h**, **i**) or in SarBC-01 only (**j**, **k**). IC_50_ and GR_50_ values for all tested compounds are reported in Supplementary Data 3.

To further define the molecular profile of the SarBC-01 model, we performed whole exome sequencing (WES) analysis in the distinct components of the patient tumor (SARC and UroCa) and derived organoids at early and late passage (passage 6 and 20, respectively). WES highlighted a close relationship between all the analyzed samples, with 231 unique alterations shared by all specimens (Sarc, UroCa, Organoids passage 6 and 20; Fig. 3a and Supplementary Data 1). Among these, we identified clonal somatic pathogenic mutations in genes that are frequently altered in sarcomatoid tumors from various epithelial entities including bladder, such as *TP53*, *RB1*, and *KRAS*^4,12^ (Fig. 3b, Supplementary Fig. 3 and Supplementary Data 1). Additional common alterations included mutations and copy number changes in epigenetic regulators (e.g., *KMT2C*, *KMT2D)*, and in EMT-associated genes (e.g., *ZEB1*, *CDH1)* (Fig. 3b).

In comparison, luminal-like UroBC-01 organoids harbored pathogenic mutations in several bladder cancer-associated genes such as *TP53*, *TSC1*, and *ELF3*, which were also found in their parental tumor (Supplementary Fig. 4 and Supplementary Data 1). Noteworthy, for both UroBC-01 and SarBC-01, main genomic drivers were clonal in all matched samples (i.e., tumor and organoids derived from same patient), while genomic subclones were detected at lesser cancer cell fractions in specific samples, highlighting intragenomic heterogeneity in tumor and organoid samples (Supplementary Fig. 5).

Overall, these results highlight the close genomic relationship between UroCa and SARC concomitant components; importantly, identified genomic drivers are conserved in SarBC-01 organoids and are enriched in sarcomatoid malignancies.

### High-throughput screening identifies drug candidates targeting SarBC-01 and UroBC-01 organoids

Given that the SarBC-01 model displayed phenotypic and genomic features reminiscent of SARC, we sought to further exploit it to identify tailored therapeutic candidates. Following optimization (Supplementary Fig. 6), we performed a high-throughput drug screening using a library comprising 1110 FDA-approved and 457 clinical compounds in both SarBC-01 and the phenotypically-distinct UroBC-01 luminal-like organoid line (1 µM concentration in triplicates; Fig. 3c; see complete list of drugs in Supplementary Data 2). These analyses led to the identification of drug candidates inhibiting UroBC-01 cells exclusively (*n* = 53 hits, group 6), SarBC-01 cells exclusively (*n* = 34 hits, group 2), or both (*n* = 70 hits, group 3) (Fig. 3d, e and Supplementary Data 2).

A subset of these drug candidates (*n* = 21), selected among standard-of-care and top hits in each group, was further tested at eight different concentrations to generate dose-response curves and determine the sensitivity profiles of UroBC-01 and SarBC-01 cells for each drug (Fig. 3f–k and Supplementary Fig. 7). Notably, although at different levels, both cell models were sensitive to chemotherapeutic drugs routinely used to treat bladder cancer (e.g., cisplatin, epirubicin, gemcitabine); as an example, the half-maximal inhibitory concentration value (IC_50_) for epirubicin, was tenfold higher in SarBC-01 cells as compared to UroBC-01 cells (IC_50=_ 0.067 µM vs. 0.006; see complete list of IC_50_ and half normalized growth rate inhibition (GR_50_)^13^ in Supplementary Data 3 and Fig. 3f); in contrast, the IC_50_ of both models was comparable for gemcitabine (IC_50=_ 0.006 µM vs. 0.005 µM) but residual live cells persisted upon treatment, even at high concentrations (Fig. 3g). Finally, although cisplatin was not identified as a hit in the screening (1 µM), a response was observed at high concentrations (IC_50=_ 7.4 µM vs. 36.6 µM; Supplementary Fig. 7a). Top hits for both models were enriched in compounds affecting the mTOR pathway, heat shock proteins, and microtubules (Fig. 3e, Supplementary Fig. 7, and Supplementary Data 2). Interestingly, the most effective hits comprised several proteasome inhibitors such as bortezomib (Fig. 3h), previously suggested to represent an attractive target in bladder cancer and other sarcomatoid tumors^14^. Additional top hits for both included compounds currently tested in clinical trials such as dacinostat, fimepinostat, and BGT-226, as well as the FDA-approved chemotherapeutic agent idarubicin (Fig. 3i and Supplementary Fig. 7). Specific targeted compounds were found to be active in only one of the models, likely reflecting their unique molecular profiles. In particular, compounds targeting tyrosine kinase receptors and the MAP kinases cascade exclusively targeted urothelial UroBC-01 cells, while drugs affecting P53 and AKT pathways (e.g., RITA and ipatasertib) were specifically efficient against sarcomatoid SarBC-01 cells (Fig. 3j and Supplementary Fig. 7e). In addition, although it was identified as a hit for SarBC-01 in the initial screen, both SarBC-01 and UroBC-01 exhibited sensitivity for the histone deacetylase inhibitor vorinostat in dose-response experiments (Fig. 3k and Supplementary Fig. 7). Among the 21 drugs tested in dose response in SarBC-01 and UroBC-01 models, six were also tested in UroBC-16 and UroBC-22 organoids (epirubicin, bortezomib, dacinostat, gemcitabine, RITA, vorinostat; Supplementary Fig. 8a and Supplementary Data 3). These analyses highlighted drug response profiles largely comparable to UroBC-01, including the presence of residual live cells upon Gemcitabine treatment and the lack of response towards RITA in contrast to SarBC-01.

### High expression of EMT-associated factors and glucocorticoid receptor are hallmarks of SARC tumors

**Fig. 4:**
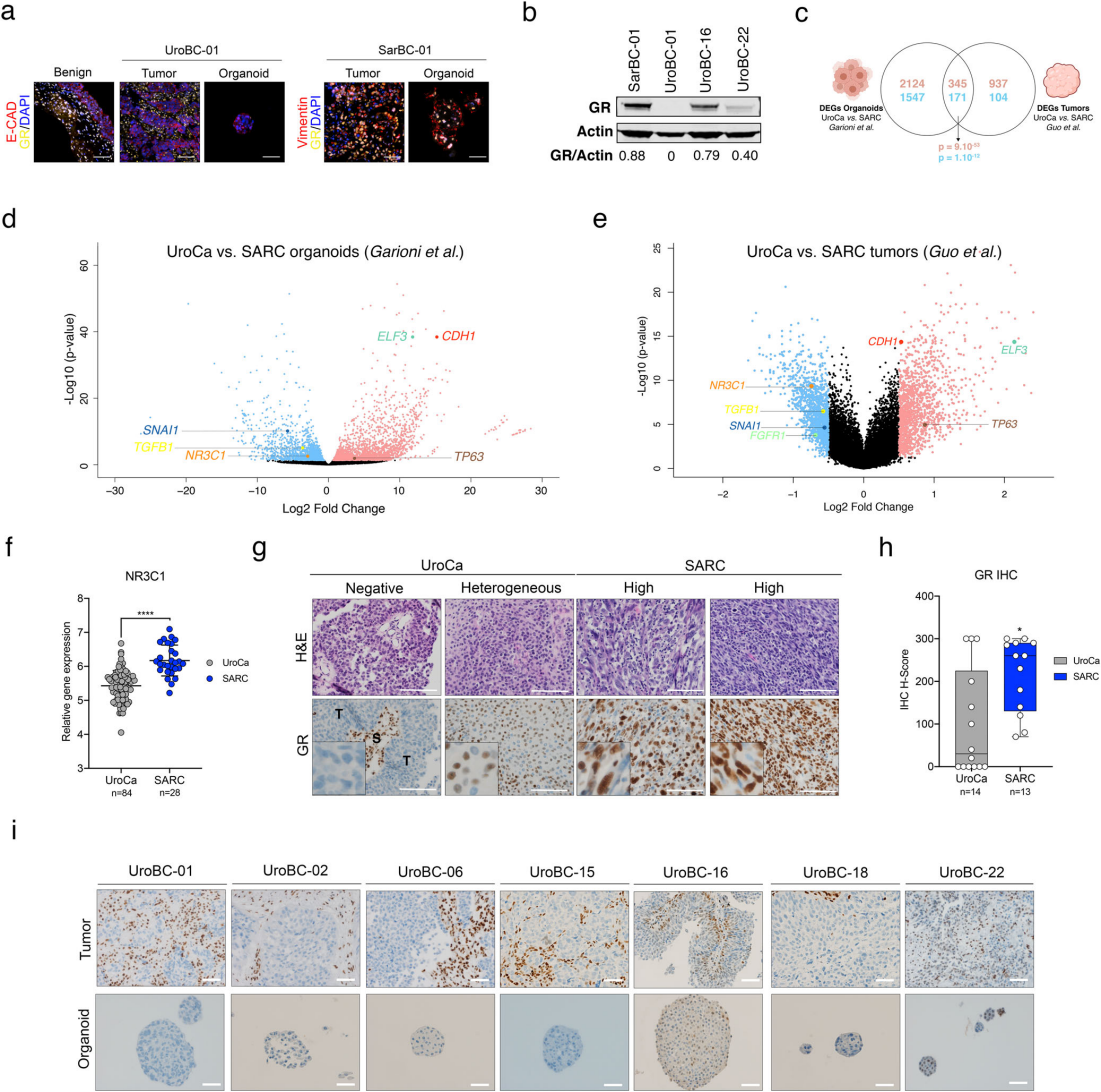
Glucocorticoid Receptor (GR) is highly expressed in SARC. **a** Immunofluorescence analyses of UroBC-01 (left) and SarBC-01 (right) tumor and organoids pairs. Shown are representative images for the indicated antibodies. GR glucocorticoid receptor, E-cad E-cadherin, Vim Vimentin, DAPI 4’,6-diamidino-2-phenylindole. Scale bars represent 50 μm. **b** Western blot analysis of GR expression in SarBC-01 (passage 73), UroBC-01 (passage 72), UroBC-16 (passage 18) and UroBC-22 (passage 8). **c** Venn diagram depicting differentially expressed genes in UroCa vs. SARC organoids and UroCa vs. SARC primary tissues. Numbers in red represent upregulated genes while numbers in blue represent downregulated genes. Overlaps’ *P* values of both upregulated and downregulated genes are shown. Statistical analysis was performed using a one-sided Fisher’s exact test. **d** Volcano plot depicting differential gene expression between UroCa organoids (*n* = 3) and SARC organoids (*n* = 3). Indicated are selected genes significantly negatively or positively enriched in UroCa models vs. SarBC-01. **e** Volcano plot depicting differential gene expression between UroCa tumors (*n* = 84) and SARC tumors (*n* = 28) as analyzed from the dataset of ref. ^4^. Indicated are selected genes significantly negatively or positively enriched in UroCa vs. SARC. **f** Expression of the *NR3C1* gene in UroCa and SARC samples. Statistical analysis was performed using an unpaired *t* test (****P < 0.001). **g** Examples of H&E and immunohistochemical staining for GR in UroCa samples and SARC samples. S Stroma, T tumor. Scale bars represent 100 μm. **h** Box plot showing significant higher frequency of positivity and higher H-score for GR in SARC (*n* = 13) vs. UroCa (*n* = 14) samples (*P < 0.05 for frequency and expression, Fisher’s exact test and Mann–Whitney test, respectively). **i** Immunohistochemistry analyses of GR expression in tumor and paired organoids for seven UroCa samples. Scale bars represent 50 µm.

In the high-throughput screen, drugs with a mode of action linked to the glucocorticoid receptor (GR) pathway were not effective in the UroBC-01 model but were among the top categories effective in the SarBC-01 model (Fig. 3e). These drugs exclusively included GR agonists such as glucocorticoids (Fig. 3e and Supplementary Data 2). Prompted by this finding, we investigated the expression of GR in UroBC-01 and SarBC-01 tumors and derived models. Consistent with a specific response of SarBC-01 cells to GR-associated drugs, GR was highly expressed in SarBC-01 tumor cells; in contrast, its expression was restricted to stromal cells in the UroBC-01 tissue sample and absent in its derived tumor organoids (Fig. 4a). These distinct levels of expression were confirmed in quantitative western blot assays, which also highlighted different degrees of expression of GR protein in UroBC-16 and UroBC-22 organoids (Fig. 4b).

To assess the broader relevance of GR expression and of our models for SARC and UroCa, we performed differential gene expression analysis in UroCa vs. SARC organoids using previously generated transcriptomic profiles (Fig. 2e). These analyses revealed 2469 upregulated genes and 1718 downregulated genes in UroCa vs. SARC models (Fig. 4c). We next analyzed a published dataset comprising transcriptomic data generated from 84 UroCa and 28 SARC samples, which revealed 1282 upregulated and 275 downregulated genes in UroCa vs. SARC tumor samples^4^. Among differentially expressed genes (DEGs) in UroCa vs. SARC, we observed a significant overlap between genes identified in organoids and genes identified in the patient tumor cohort (345 genes up, 171 genes down; *P* = 9.10^-53^ and *P* = 1.10^12^, respectively, one-sided Fisher’s exact test; Fig. 4c). In both datasets, among the overlapping top genes positively enriched in UroCa, were the gene encoding E-cadherin (*CDH1*) and other epithelial-associated genes such as *ELF3* and *TP63* (Fig. 4d, e). The top overlapping genes negatively enriched in UroCa vs. SARC included genes associated with EMT (e.g., *SNAI1*, *TGFB1*); in line with these data, unsupervised clustering analysis of the two datasets using an EMT gene signature^15^ distinguished UroCa and SARC-derived sample groups (Fig. 4d, e and Supplementary Figs. 9 and 10). Notably, the gene encoding GR (*NR3C1*) was among the genes significantly more expressed in SARC vs. UroCa samples in both the organoid and tumor sample cohorts (*P* < 0.0001, unpaired *t* test; Fig. 4d–f). Taken together, these data suggest that organoid models emulate key transcriptomic features of patient tumors. In addition, the expression of EMT-associated genes and of *NR3C1/*GR appears to be enriched in SARC.

To validate these findings at the protein level and in another independent cohort, we assessed GR expression via immunohistochemistry in an in-house cohort comprising 14 UroCa and 13 SARC paraffin-embedded samples (Supplementary Data 4). In the UroCa group, GR expression was heterogenous with a large subset of negative/low tumors (*n* = 8/14 samples with H-score <50) and a minor subset exhibiting high expression (*n* = 3/14 with H-score of 300). To note, two out of three UroCa samples with high GR expression were diagnosed as “Basal/Squamous”. SARC tumor samples displayed significantly higher frequency of positivity (*n* = 13/13 positive SARC) and higher H-score of GR, as compared to UroCa samples (*P* = 0.02 Fisher’s exact test, *P* = 0.03 Mann–Whitney test, respectively; Fig. 4g, h, Supplementary Fig. 11, and Supplementary Data 4). Finally, absence/low expression of GR in UroCa samples was recapitulated in matched organoids, further highlighting the relevance of those models to study BC (Fig. 4i). In line with our data in human BC, *nr3c1* was highly expressed in basal/squamous specimens and higher in sarcomatoid samples, as compared to benign-like, hyperplasia, dysplasia and low-grade UroCa samples derived from a mouse model of bladder cancer (ref. ^16^ and Supplementary Fig. 12).

### Glucocorticoid treatment leads to morphological and transcriptomic changes associated with EMT reversion

**Fig. 5:**
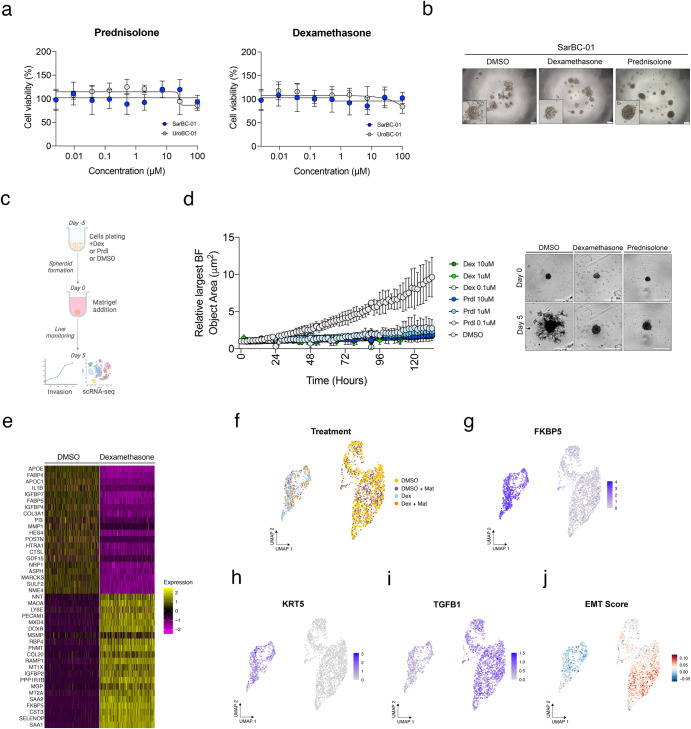
Glucocorticoid treatment leads to morphological and transcriptomic changes associated with reversion of epithelial-to-mesenchymal transition. **a** Glucocorticoids such as prednisolone and dexamethasone have no significant effect on the viability of SarBC-01 cells in dose response experiments. **b** Change of morphology of SarBC-01 upon treatment with 1 µM of dexamethasone or 1 µM of prednisolone. Scale bars represent 200 μm. **c** Schematic representation of the workflow followed for the in vitro invasion assay and single-cell RNA sequencing. **d** Left: Largest bright field object area measured over time for spheroids generated from SarBC-01 cells treated with three concentrations of dexamethasone (Dex) or prednisolone (Prdl) as compared to the DMSO control. Analyses were performed using a mixed-effects analysis (*P* = 0.0001). Data are represented as means of three spheroids (relative to day 0 for each) and the error bars represent standard deviations (SD). Two independent biological replicates were performed and one representative replicate is shown. Right: Representative images of SarBC-01 spheroids treated with DMSO, 0.1 µM of dexamethasone or 0.1 µM of prednisolone at day 5 and day 10 are shown. Scale bars represent 800 µm. **e** Heatmap displaying top 20 up- and downregulated genes in dexamethasone- vs DMSO-treated SarBC-01 spheroids after random down-sampling of DMSO-treated condition. **f**–**j** Single-cell RNA sequencing of SarBC-01 cells (passage 52) following dexamethasone treatment. 2170 cells were analyzed following incubation with DMSO or dexamethasone in invasion assay conditions and in presence of absence of Matrigel, leading to four different culture conditions: DMSO: 1071 cells; DMSO + Mat: 501 cells; Dex: 442 cells; Dex + Mat: 156 cells. Shown are UMAP representations of the treatment conditions (**f**), expression of selected indicated genes (**g**–**i**) and aggregated EMT score (**j**).

Prompted by these findings and by the drug screen results, we next sought to assess the effects of glucocorticoids (GC) in dose response experiments. In particular, we focused on prednisolone (Prdl) and dexamethasone (Dex), which are routinely administered as adjuvant therapy to manage side effects caused by anticancer therapy in patients with solid tumors. While they were both identified as hits for SarBC-01 organoids in the high-throughput drug screen (52.26 and 51.55% of growth inhibition respectively), the efficacy of Prdl and Dex was not consistently confirmed in dose response experiments (Fig. 5a and Supplementary Fig. 8b). A notable change of morphology was, however, observed in SarBC-01 cells upon treatment with Prdl or Dex (Fig. 5b). In line with these observations, the invasive ability of SarBC-01 cells was significantly reduced upon treatment with those drugs, even at low concentrations mirroring plasma levels in patients (0.1 µM Dex or Prdl vs. DMSO, mixed-effects analysis, *P* < 0.0001; Fig. 5c, d and Supplementary Videos 5 and 6). To test whether GC-induced phenotypic effects on organoids correlated with transcriptomic changes at the cellular level, we performed single-cell RNA sequencing (scRNA-seq) in SarBC-01 cells treated with 0.1 µM Dex or with DMSO in invasion assay conditions. In order to account for potential effects of the Matrigel on the transcriptomic profile of the cells, the experiment was performed with and without Matrigel, leading to 4 distinct conditions (DMSO, DMSO + Matrigel, Dex, and Dex + Matrigel); prior to sequencing, these 4 conditions were multiplexed using MULTI-seq lipid-tagged indices, allowing to minimize technical confounders such as doublets and batch effects^17,18^ (see “Methods” and Supplementary Fig. 13). A total of 2170 cells was retrieved from the distinct conditions (median of 6700 genes per cell), which clustered based on treatment conditions and largely independently from the presence of Matrigel (Fig. 5e).

Notably, genes associated with metastasis, migration and invasion were among the top downregulated genes in Dex-treated SarBC-01 cells (Fig. 5e and Supplementary Fig. 14). Those included *COL3A1* (Collagen Type III Alpha 1 chain), *MMP1* (Matrix Metalloprotein 1), *CTSL* (Cathepsin L), *NRP1* (Neuropilin 1), and *POSTN* (Periostin) among others. As expected, cells treated with Dex displayed significantly higher levels of *FKBP5*, a GR target gene whose transcription is induced upon GR transactivation (Fig. 5g^19^). Dex-treated cells were also characterized by gained expression of the two urothelial markers *KRT5* and *KRT7*, suggesting that SARC cells acquired epithelial-like transcriptomic features upon Dex treatment (Fig. 5h and Supplementary Fig. 15). Conversely, Dex-treated cells displayed significantly lower levels of genes associated with EMT such as *TGFB1*, *FGF2*, *SNAI2*, *IL1B*, *WNT5A*, *NOTCH1* and *VEGFA*, as compared to the DMSO condition (Fig. 5i and Supplementary Fig. 15). Consistent with these data, cells treated with Dex associated with a significantly lower EMT score as compared to cells treated with the DMSO control (Fig. 5j, score calculated based on a list of genes previously reported as EMT markers^15^).

Collectively, these data show that SARC cells gain epithelial-like features and lose mesenchymal-like features upon treatment with GC, suggesting that the SARC phenotype is plastic and may be therapeutically modulated.

## Discussion

A lack of experimental models emulating rare tumor entities, such as SARC, hampers the efforts to decipher mechanisms driving such diseases and the development of tailored clinical strategies. In this study, we address this limitation by establishing a 3D multicellular in vitro model derived from a SARC patient that retains key phenotypical and molecular features of SARC and is tumorigenic in vivo. While few SARC-like models have recently been described^20,21^, SarBC-01 represents the first fully characterized long-term organoid model derived from a SARC patient; such type of model better recapitulates heterogeneity than cell lines and is more easily amenable to drug screen than xenografts, making it an attractive model for basic and translational research. Although the phenotype of SarBC-01 remained largely stable over time, we observed some phenotypic and genomic changes between early- and late-passage SarBC-01 organoids, with late-passage organoids seemingly better mirroring features of SARC (Figs. 2 and 3 and Supplementary Fig. 5). These changes may reflect culture-induced tumor plasticity, a phenomenon which may occur upon long-term culture of bladder cancer PDOs and may result in more aggressive phenotypes^10^. Taken together, these observations underline the importance of frequently monitoring features of novel long-term organoid models and re-emphasize their potential to emulate tumor evolution in vitro.

High-throughput drug screening highlighted the efficacy of standard chemotherapies, as exemplified by epirubicin which was active in SARC and UroCa organoids. Further, these analyses revealed potential novel therapeutic options displaying higher efficacy as compared to the standard of care, such as the proteasome inhibitor bortezomib and the p53 interacting molecule RITA. Importantly, the SarBC-01 model is derived from one single patient and we acknowledge that the identification of specific targeted compounds may reflect its unique genomic profile rather than its sarcomatoid identity. Nevertheless, SarBC-01 cells harbor mutations in genes that are commonly altered in a broad range of tumors and enriched in sarcomatoid tumors^4,12^, and exhibit transcriptomic and phenotypical features typical of SARC, making it a model that could be broadly generalized to SARC. The direct translatability of our findings to patient therapies is also limited by the use of single drugs in our screen, while combination therapies are currently favored in the clinical setting. Nonetheless, our findings provide a guidance for the selection of compounds that may have favorable outcome if tested in novel clinical trials.

Although their effect on viability was modest, drugs with a mode of action linked to the glucocorticoid receptor (GR) pathway were among the top categories effective in the SarBC-01 model in the high-throughput screening. These data led us to investigate the expression of the steroid hormone receptor GR in tumors samples obtained from UroCa and SARC. Exploiting two distinct cohorts, we showed that high *NR3C1*/GR expression is a feature characterizing a large subset of SARC tumors. In contrast, UroCa tumors harboring a predominantly luminal phenotype tended to express low levels of *NR3C1*/GR, a feature which is conserved in matched organoids. While GR signaling has not been extensively studied in the context of bladder cancer, limited available data have pointed to a multifaceted and context-specific role of this pathway^19,22,23^. In UroCa, GR expression has been shown to be lower in high-grade *vs*. low-grade as well as in muscle-invasive *vs*. non-muscle-invasive disease, consistent with a potential tumor-suppressor function^19^. Noteworthily, while we observed a large subset of muscle-invasive UroCa tumors with negative GR, two out of three UroCa samples displaying high GR expression were classified as “Basal/Squamous”, a subtype from which SARC has been proposed to originate from^4^. In contrast to UroCa, the relevance of GR and its associated pathway had not been addressed in the context of histological variants of bladder cancer, such as SARC so far. Noteworthily, other sarcomatoid tumor entities have been suggested to display high GR levels, suggesting that our findings may have relevance beyond the bladder context^24^.

Finally, we show that GC treatment induces epithelial-like morphological and transcriptomic changes which are consistent with (partial) reversion of EMT in SARC cells (*See hypothetical model* in Supplementary Fig. 16). These data suggest a potential benefit of GC compounds which are commonly used in an adjuvant and palliative setting in various tumor types^25^. Notably, GR activation has been shown to contribute to both EMT and mesenchymal-to-epithelial transition, depending on the tissue type and the context^26,27^. Thus, given the dual action of GC in cancer and the complex role of GR signaling in bladder carcinogenesis^19,27^, further investigations are warranted to investigate mechanisms driving their effects. Accounting for GR debated role in BC, a precise assessment of the histological subtype and of GR expression in patients’ tumor samples may allow informed decision regarding the use of GC.

Collectively, our data highlight the high plasticity potential of the SARC phenotype. We first show that the SARC tumor and its derived cells display a high degree of genomic similarity with their UroCa counterpart, suggesting a common ancestor as previously proposed^4,8^. The absence of relevant private genomic alterations in the SARC counterpart potentially explaining the evolution of the cells from UroCa to SARC and the partial recovery of epithelial features in xenografts, raise the hypothesis that SARC differentiation might be a reversible phenomenon driven by microenvironmental cues. Finally, the EMT reversal effects associated with GC treatment further emphasize the plasticity of the SARC phenotype and suggest that it may be therapeutically modulated towards less aggressive phenotypes.

Altogether, our study highlights the power of organoid models to identify precision oncology strategies for rare tumor entities and to provide key insights into factors driving these diseases.

## Methods

### Patients and clinical samples

Bladder cancer samples used for organoid generation were obtained from patients operated at the University Hospital of Basel (USB) following written informed consent under an approval by the Ethical Committee of Northwestern and Central Switzerland (EKBB 37/13).

In addition, immunohistochemical staining and scoring for GR expression was performed on an archival cohort of 14 UroCa samples and 13 SARC samples under an approval by the Ethical Committee of Northwestern and Central Switzerland (EKNZ 2014-313). Clinical and pathological characteristics of the two sample cohorts are described in Supplementary Data 4.

### In vitro model generation

Bladder cancer tissues were washed in aDMEM/F12 (GIBCO, 12634028). One portion of the sample was fixed in formalin 4% and kept at 4 °C until further processing. The rest of the sample was processed as described in ref. ^10^. Briefly, the tissue was mechanically and enzymatically digested, and filtered through a 100 µm strainer (Corning, 431752) to generate a cell suspension. Cells were counted and resuspended in order to reach a concentration of 5 × 10^3^ cells/well in a 70/30 ratio Matrigel (Corning, 356231)/organoid culture medium. The organoid culture medium was comprised of aDMEM/F12 supplemented with 100 ng/ml WNT3A (Bio-Techne, 5036-WN), 100 ng/ml R-Spondin1 (R&D Systems, 4645-RS), 50 ng/ml EGF (Peprotech, AF-100-15), 1mM N-acetyl-L-cysteine (Thermo Fisher, A15409.36), 100 ng/ml Noggin (Peprotech, 120-10C), 1 µM TGFβ inhibitor (Selleck, LY2157299), 1× N2 (GIBCO, 17502048), 1× B27 solution (GIBCO, 17504044), 10 µM Y-27632 (AbMole Bioscience, M1817) (adapted from ref. ^28^). Subsequently, cells were plated in a 10 µL drop at the bottom of the well of a 96-wells plate and incubated at 37 °C and 5% CO_2_ for 30 min, resulting in the embedment of the cells in a solid Matrigel drop (embedded condition; EMB). 100 µL of organoid culture medium was added to the wells and fresh medium was dispensed every 3–4 days; organoids were maintained for a maximum of 30 days in culture before passaging at a 1:2 dilution. For passaging, Matrigel was digested by adding 1 mg/ml dispase (GIBCO,17105041) solution to the wells and incubating for 60 min at 37 °C before harvesting. The collected organoids underwent enzymatical digestion to generate a suspension of cells, that were re-seeded in EMB conditions (for expansion) or resuspended in culture medium containing 5% Matrigel and plated in low-adherence plates for all the subsequent analysis (non-embedded condition; NE). All in vitro experiments were performed in NE conditions.

### Histology, immunohistochemistry and immunofluorescence

Histological analysis was performed by standard hematoxylin and eosin (H&E) staining. Immunohistochemical analyses were conducted according to standard indirect immunoperoxidase procedures. Immunofluorescence staining was performed on 4‐μm‐thick sections, following antigen retrieval with boiling citrate acid‐based antigen unmasking solution at 98 °C for 15 min such as in ref. ^29^. Immunofluorescence images were captured using a Nikon Ti2 microscope. Details of all the antibodies and dilutions are provided in Supplementary Data 5.

### Proliferation assay

To evaluate the growth rate of the generated organoid models, cells were plated in NE conditions at a concentration of 1000 cells/well in low-adherence 384-well plates (Greiner Bio-One, 7.781 976-SIN) in triplicates. Viability of the cells was monitored over 12 days by luminescence using CellTiter-Glo 3D (Promega, G9681) according to manufacturer instructions. The proliferation rate for each line was estimated using the fit easylinear function from the R package growth rate v.0.8.4 (https://github.com/tpetzoldt/growthrates). The proliferation rate was assessed at passage 69 of SarBC-01, passage 68 of UroBC-01, passage 13 of UroBC-16 and passage 5 of UroBC-22.

### Invasion assay

The invasion assay was performed as described in ref. ^30^. Briefly, single cells derived from organoids were plated in low-adherence U-shaped 96-well plates at a concentration of 1000, 500, 250, 125 cells/well in organoid culture medium. After 5–8 days in culture, spheroids formed at the bottom of the wells, and about 70% of the culture medium was carefully removed and replaced with ice-cold Matrigel. The plates were spun down at 350 g for 10 min at 4 °C and placed in the Incucyte® Live-Cell Analysis System (Sartorius). The growth of the spheroids was monitored for the following 7 days and invasion was automatically quantified via the Incucyte® Spheroid module analysis tool, measuring the increase of the total spheroids area over time. Data were normalized by the area measured at day 0 of monitoring.

To assess the invasion capacity upon glucocorticoids treatment, a similar approach was applied (Fig. 5c). Briefly, 1000 cells were plated in low-adherence U-shaped 96-wells plates and supplemented with culture medium containing dexamethasone 10 µM, 1 µM, 0.1 µM, prednisolone10 µM, 1 µM, 0.1 µM or 1% DMSO as negative control in triplicates. After 5 days in culture, about 70% of the culture medium was carefully removed and replaced with ice-cold Matrigel (day 0 monitoring). The spheroid growth was monitored via Incucyte® for the following 5 days and quantified with the Spheroid module analysis tool, measuring the increase of the spheroids area normalized by the area measured at day 0 of monitoring.

The invasion capacity was assessed at passage 40 and 50 of SarBC-01, passage 39 of UroBC-01, passage 16 of UroBC-16 and passage 9 of UroBC-22.

### In vivo tumorigenic capacity

All mouse experiments were conducted with the approval of the Animal Care Committee of the Kanton Basel-Stadt, Switzerland (3066-32428). Mice were bred and maintained in the animal facility of the Department of Biomedicine of the University Hospital Basel under specific pathogen-free conditions on a 12 h day and 12 h night schedule with ab libitum access to food and drinking water. Single-cell suspensions derived from organoids were spun down and resuspended at a 50/50 ratio Matrigel/PBS solution. One million of cells was subcutaneously injected in the flank of NOD *scid* gamma (NSG); two mice were injected per organoid line to compare the tumorigenicity capacity between the two lines (passage 32 and 31 for SarBC-01 and UroBC-01, respectively). In addition, 2 mice were injected to confirm the tumorigenic potential of SarBC-01 cells at a different passage (passage 36, 2/2 mice with tumors). Tumor size was measured twice weekly and tumors were harvested when reached 1500 mm^3^ in volume. The human origin of all patient-derived organoid xenografts (PDOXs) was confirmed by staining with an antibody recognizing human mitochondria (ab92824, Abcam).

### DNA and RNA extraction

SarBC-01 DNA was extracted from formalin-fixed paraffin-embedded (FFPE) material (SARC and UroCa, components; healthy lymph nodes for germline DNA), and flash-frozen organoids. UroBC-01 DNA was extracted from fresh peripheral blood mononucleated cells (PBMCs) for germline and from flash-frozen material for the parental tumor and derived organoids. RNA was extracted from SarBC-01, UroBC-01, UroBC-16 and UroBC-22 fresh and flash-frozen organoids.

For FFPE tissue, 10-μm thick unstained tissue sections were cut on glass slides. The distinct morphological components of sarcomatoid and urothelial bladder cancer (SARC and UroCa, respectively) were identified and marked by a trained pathologist. The samples were deparaffinized with incubation in Xylene for 5 min and the regions of interest scratched from the glass slide and collected. DNA was extracted using the RecoverAll RNA/DNA extraction kit (Invitrogen, AM1975), according to the manufacturer’s instructions. The collected DNA underwent incubation with Uracil-DNA Glycosylase at 37 °C for an hour, followed by an incubation at 50 °C for 10 min. For fresh and flash-frozen tissue, DNA and RNA were simultaneously isolated using the Quick-DNA/RNA Miniprep kit (Zymo Research, D7001), according to the manufacturer’s instructions. Flash-frozen tissues were crushed in liquid nitrogen with plastic pestels (Fisher, Thermo Fisher Scientific, 12141363) prior to isolation. DNA and RNA were quantified using the Qubit Fluorimeter assay (Thermo Fisher Scientific).

### Whole exome sequencing (WES) and variant annotation

DNA extracted from FFPE and flash-frozen specimens was subjected to WES. Twist Human Core Exome + RefSeq + Mito-Panel kit (Twist Bioscience, 102031) was used for the whole exome capturing according to manufacturer’s guidelines. Sequencing was performed on Illumina NovaSeq 6000 using paired-end 100-bp reads and yielded a mean depth of coverage comprised between 108.6× and 153.4×. Sequencing was performed by CeGaT (Tübingen, Germany) and FASTQ processing workflow was adapted from ref. ^31^. Briefly, reads were aligned to the reference human genome GRCh38. Somatic variants were detected using MuTect2^32^ and discarded if they had a variant allelic fraction <5% or if were covered by fewer than three reads. To filter out potential artifacts, we further excluded variants present in more than two of a panel of 123 non-tumor samples. The complete list of identified variants is provided in Supplementary Data 1. Variant annotation was performed by SnpEff software v.4.1^33^. The heatmap of non-synonymous mutations was generated using the R package maftools v.2.10.5^34^.

### Copy number aberration and clonal analysis

Allele-specific CNAs were identified using FACETS v.0.5.6^35^. Genes were classified using the following criteria: “gains”: total copy number greater than gene-level median ploidy; “amplification”: total copy number greater than ploidy +4; losses: total copy number less than ploidy; homozygous deletion: total copy number of 0. Loss of heterozygosity were identified as those where the lesser (minor) copy number state at the locus was 0. Clonal analysis was performed with the ABSOLUTE V2.0^36^ algorithm. Solutions from ABSOLUTE were manually curated to assure the solution matched the ploidy estimate generated by FACETS. For heatmap representations, mutations and copy number alterations were shown based on top 100 altered genes in muscle-invasive bladder cancer (TCGA study^37^), or selected genes associated with specific pathways (sarcomatoid^12^, epigenetic^38^, EMT^15^). Cancer cell fraction estimates generated by ABSOLUTE were used as input to PhylogicNDT^39^ to find mutation clusters, infer subclonal populations and their phylogenetic relationships.

### Drug screening

To evaluate the assay quality, wells were plated in NE conditions at a concentration of 250, 500, or 1000 cells/well in low-adherence 384-well plates (Greiner Bio-One, St.Gallen, Switzerland). After 5 days, 12.5 µL of organoids culture medium containing either DMSO (negative control) or staurosporine (positive control) was added and after 5 additional days, the viability of the cells was assessed by luminescence using CellTiter-Glo 3D (Promega) according to manufacturer instructions. Luminescence was read on a Synergy H1 Multi-Mode Reader (BioTek Instruments). The z’ factor for each seeding concentration was calculated as:

$${z}^{{\prime} }={\bf{1}}-\frac{{\bf{3}}* ({\boldsymbol{median}}(\left|{{\boldsymbol{ctrl}}}^{+}-{\boldsymbol{median}}({{\boldsymbol{ctrl}}}^{+})\right|)+{\boldsymbol{median}}({\boldsymbol{median}}(\left|{{\boldsymbol{ctrl}}}^{-}-{\boldsymbol{median}}({{\boldsymbol{ctrl}}}^{-})\right|))}{\left|{\boldsymbol{median}}\left({{\boldsymbol{ctrl}}}^{+}\right)-{\boldsymbol{median}}({{\boldsymbol{ctrl}}}^{-})\right|}$$

An assay was considered of good quality if it had a z’ factor above 0 and no positional effects. For the main assay, cells were plated at a concentration of 1000 cells/well. Five days after plating, 1567 compounds (1110 FDA-approved drugs and 457 in clinical trial drugs, obtained from NEXUS Personalized Health Technologies, Zürich, Switzerland) were added in triplicates at a 1 µM concentration. After 5 days of treatment, the viability of the organoids was measured as described above and normalized to the negative control); The normalized percentage inhibition (NPI) was calculated for each drug as:

$${NPI}=\frac{{\boldsymbol{median}}\left({{\boldsymbol{ctrl}}}^{-}\right)-{\boldsymbol{sample}}}{{\boldsymbol{median}}({{\boldsymbol{ctrl}}}^{-})-{\boldsymbol{median}}({{\boldsymbol{ctrl}}}^{+})}$$

Compounds were defined as “hits”, if the median of the NPI was higher than 0.5. Compounds were defined as “anti-hits”, if the median of the NPI was lower than −0.5 (Supplementary Data 2).

### Drug-dose response analysis

To validate results from the drug screen, cells were plated at a concentration of 1000 cells/ well in low-adherence 384-well plates. 23 drugs from the identified hits were selected and an 8-point dilution series of each compound was dispensed in triplicate using a Tecan Digital Dispenser D300e (Tecan). Drug concentrations spanned from 10 pM to 1 mM, depending on the drug. Cell viability was measured by CellTiter-Glo 3D assay following 5 days of drug incubation, and results were normalized to untreated controls. Data analyses were performed using GraphPad Prism, and the values of IC_50_ (see Supplementary Data 3), Hill slope, and AUC were calculated by applying nonlinear regression (curve fit) and the equation log(inhibitor) vs. normalized response (variable slope). The IC_50_ value was further normalized accounting for the proliferation rate of each organoid model using the Growth Rate Inhibition metric (GR_50_) (ref. ^13^ and Supplementary Data 3).

### In silico mRNA expression analysis

Public datasets were downloaded from the GEO database (Human dataset: accession number GSE128192; mice dataset: accession number GSE197016). The human dataset comprised 28 cases of SARC and 84 cases of UroCa^4^. Differential gene expression analysis on this dataset was performed using the GEO2R online tool with default parameters. In brief, comparison between SARC and UroCa samples was carried out using the limma package v.3.52.4 and the *P* value were adjusted using the Benjamini–Hochberg correction. Genes with FDR–adjusted *P* values < 0.05 and fold changes >0.5 were considered as differentially expressed. The comparison of *NR3C1* expression levels was carried out using an unpaired *t* test. The mouse dataset comprised 37 bladder cancer samples deriving from mice exposed to N-butyl-N-(4-hydroxybutyl)-nitrosamine (BBN)^16^. The data were log-normalized and the samples classified and ordered based on their pathological stage, as described in the original publication. *Nr3c1* expression levels were plotted for each sample class.

### Bulk RNA sequencing

RNA extracted from fresh and flash-frozen samples was subjected to bulk RNA sequencing. TruSeq Stranded mRNA kit (Illumina, 20020594) was used for the library preparation according to manufacturer’s guidelines. Sequencing was performed on Illumina NovaSeq 6000 using paired-end 100-bp reads. Sequencing was performed by CeGaT. The resulting FASTQ were trimmed using Trimmomatic V.0.39^40^ and aligned to the GRCh38 human reference genome using STAR V2.7.9a^41^. Transcript quantification was performed using RSEM V.1.3.3 (PMID:21816040). For subsequent analyses, genes that were expressed in less than 2 samples were discarded and counts were normalized using DESeq2 V3.17^42^. Principal Component Analysis was run on all the sequenced samples. To generate heatmaps, means were subtracted from each gene of the log-transformed count matrix. Heatmaps and unsupervised clustering of genes and samples were performed with pheatmap V1.0.12 (https://github.com/raivokolde/pheatmap). Differential Gene Expression analysis was performed with DESeq2. Genes with adjusted *P* values < 0.05 and fold changes >0.5 were considered as differentially expressed. The significance of overlapping differentially expressed genes between this dataset and the Guo et al. public dataset (2) was calculated with a one-sided Fisher’s exact test. Bulk RNA sequencing analysis was performed at passage 6, 19 and 59 of SarBC-01, passage 70 of UroBC-01, passage 19 of UroBC-16 and passage 8 of UroBC-22.

### GR protein expression analysis

For IHC analysis, the antibody against GR (ab183127, Abcam) was tested on sections from formalin-fixed paraffin-embedded kidney, testis, and pancreas positive controls and assessed by a trained pathologist. A composite scoring system (H-score) was used by multiplying a given nuclear intensity (between 0 and 3) by the percentage of positive cells. Details of the patients’ cohorts and staining results can be found in Supplementary Data 4.

Western blot analysis was performed as previously described^43^. In brief, total proteins were extracted by cell lysis and quantified with a Protein Assay Kit II (Bio-Rad, 5000002). In total, 10 µg of proteins were loaded onto a NuPAGE 10% Bis-Tris gel (Invitrogen, NP0306BOX) for electrophoresis. Proteins were transferred to a nitrocellulose membrane using the Trans-Blot Turbo Transfer System (Bio-Rad) and probed with primary antibodies. Next, the membranes were incubated with fluorescent secondary antibodies and scanned using the Odyssey Infrared Imaging System (LI-COR Biosciences). Proteins were quantified using ImageJ^44^ and expression of GR was normalized with β-actin. Unprocessed blots are provided in Supplementary Fig. 17.

### Single-cell RNA-sequencing (scRNA-seq) sample preparation and multiplexing

SarBC-01 cells derived from organoids were plated in low-adherence U-shaped 96-wells plates at a concentration of 2000 cells/well and supplemented with medium containing either 100 nM Dexamethasone (Dex) or 1% DMSO as negative control. After 5 days in culture, spheroids formed at the bottom of the wells, and half of the culture medium was carefully removed and replaced either with ice-cold Matrigel (Mat) or freshly prepared medium, resulting in four different culture conditions ( + Mat/Dex, +Mat/DMSO, −Mat/Dex, −Mat/DMSO). After 5 days, the spheroids were harvested upon incubation with TrypLe for 15 min, dissociated to single cells by gentle pipetting and washed in PBS. Cells belonging to the distinct culture conditions were subsequently processed for multiplexing using the MULTI-seq protocol^17^. Briefly, a mix of lipid-modified DNA oligonucleotides and unique barcode oligonucleotides for each culture condition was added to the cells and incubated in cold PBS for 5 min. Next, a lipid-modified co-anchor was added to each sample to stabilize the membrane-bound barcodes. After a 5-min incubation on ice, cells were washed in PBS containing 1% FBS 1% BSA to quench unbound barcodes. Cell number and cell viability was then assessed for each sample with an automatic hematocytometer. Finally, samples were pooled together with comparable cell number, washed with PBS 1% FBS 1% BSA, and 15 000 cells were loaded in a Chromium Single Cell 3ʹ GEM Library and Gel Bead Kit v3 (10x Genomics).

### scRNA-seq library preparation, sequencing, and quality control

Gene expression (cDNA) and MULTI-seq libraries were prepared according to the manufacturer’s protocol of Chromium Next GEM Single Cell 3’ reagents Kits v3.1 (Dual Index). In brief, after GEMs embedding, cDNA was generated by reverse transcription reactions. MULTI-seq barcode fragments were separated from endogenous cDNA fragments during the first round of size selection using SPRIselect beads (Beckman Coulter, B23317). Next, cDNA and MULTI-seq fragments were processed separately. Fragmentation, end repair, and A-tailing procedure were performed on endogenous cDNA fragments, and sample dual indexes (Dual Index TT Set A plate) were lastly added over PCa amplification. After clean-up, MULTI-seq barcode fragments were PCR amplified and tagged with RPI index (TruSeq technology) and i5 universal index. Both libraries were cleaned up with SPRIselect beads to avoid primer contamination and fragments with inappropriate size^17^. cDNA and MULTI-seq libraries were analyzed using an Agilent Bioanalyzer (DNA High Sensitivity kit) prior to sequencing on a NovaSeq6000 (S2 flow cell) platform. Cellranger (v.6.1.2) was used to process the cDNA raw data and generate a raw count matrix that was subsequently loaded in the R package Seurat (v.4.3.0)^45^. Sample barcodes were demultiplexed using the HTODemux function implemented in Seurat^17^. Cells labelled as doublet or negative were removed from the Seurat object. Furthermore, cells with less than 2500 detected genes and more than 25% mitochondrial transcripts were filtered out. A total of 2170 cells was obtained and subsequently analyzed.

### scRNA-seq bioinformatic analysis

The Seurat dataset was log normalized and 2000 variable features were initially computed using the ‘vst’ method. Data were scaled and the first 30 principal components were calculated for downstream analyses. Uniform Manifold Approximation and Projection (UMAP)^46^ used for visualization. Cell clusters that were including the cells within the different culture conditions were identified with the ‘FindClusters’ function offered by Seurat with a resolution of 0.8. Exploratory analysis was performed to visualize the expression of genes linked to glucocorticoid receptor pathway and EMT. The EMT score was calculated with the ‘AddModuleScore’ function, using a list of genes previously reported as EMT markers^15^. Differential gene expression analysis was performed with the MAST method^47^ implemented in Seurat within the ‘FindMarkers’ function. The analysis was limited to genes detected in at least 25% of the tested populations and showing at least ±0.5 log fold change difference.

### Statistical analysis and data representation

The Prism GraphPad software (version 8.0, San Diego, CA, USA) was used for statistical analyses and data representation. Statistical analysis of invasion assays was conducted by mixed-levels analysis. The comparison of *NR3C1* mRNA levels between UroCa and SARC was conducted by an unpaired *t* test. To define overlapping differentially expressed genes between our bulk RNA seq dataset and the Guo dataset, one-sided Fisher’s exact test was used. For GR protein expression, the average of the H-Score was compared with a Mann–Whitney test, while the frequency of positivity in each group was assessed using a Fisher’s exact test. For all statistical analyses, *P* values < 0.05 were considered significant.

### Reporting summary

Further information on research design is available in the Nature Research Reporting Summary linked to this article.

### Supplementary information

Supplementary Material

Supplementary Data 1

Supplementary Data 2

Supplementary Data 3

Supplementary Data 4

Supplementary Data 5

In vitro invasion assay SarBC-01.

In vitro invasion assay UroBC-01.

In vitro invasion assay UroBC-16.

In vitro invasion assay UroBC-22.

In vitro invasion assay SarBC-01 (DMSO treated).

In vitro invasion assay SarBC-01 (Dexamethasone treated).

REPORTING SUMMARY

## Data Availability

The whole exome sequencing, single-cell RNA sequencing, and mRNA sequencing data generated in this study are available under restricted access at the European Genome-phenome Archive (accession EGAS00001007430). The use of the data will be subjected to agreement of a data use policy which details the minimum protection measures required to data encryption and user access.

## Code availability

No custom code has been used in this publication. All the data processing steps along with R packages versions and scripts used are described in “Methods”.
