# Supplementary material for: Patient-derived organoids identify tailored therapeutic options and determinants of plasticity in sarcomatoid urothelial bladder cancer
Source: NPJ Precis Oncol. 2023 Nov 2;7:112. doi: 10.1038/s41698-023-00466-w (PMC10622543; doi:10.1038/s41698-023-00466-w)
Supplement: Supplementary file 1 — Supplementary Material [file 41698_2023_466_MOESM1_ESM.pdf]

Patient-derived organoids identify tailored therapeutic options and determinants of plasticity in sarcomatoid urothelial bladder cancer

Supplementary Material List

Supplementary Figures

Supplementary Figure 1. Characterization of organoid models

Supplementary Figure 2. Characterization of SarBC-01 and UroBC-01 Patient-Derived Organoid Xenografts (PDOXs)

Supplementary Figure 3. Frequency of specific genomic alterations in sarcomatoid tumors

Supplementary Figure 4. Whole Exome Sequencing Analysis of UroBC-01 tumor and matched organoids

Supplementary Figure 5. Clonal evolution in SarBC-01 and UroBC-01 tumors and derived models

Supplementary Figure 6. Optimization and quality control of the high-throughput drug screen

Supplementary Figure 7. Drug response analysis for additional selected compounds in SarBC-01 and UroBC-01

Supplementary Figure 8. Drug response analysis for selected compounds in UroBC-16 and UroBC-22

Supplementary Figure 9. Expression of EMT-associated genes in SARC vs. UroCa organoids

Supplementary Figure 10. Expression of EMT-associated genes in SARC vs. UroCa tumors

Supplementary Figure 11. Glucocorticoid Receptor (GR) expression in UroCa and SARC samples

Supplementary Figure 12. Expression of *Nr3c1* in a mouse model of bladder cancer progression

Supplementary Figure 13. scRNA-seq quality controls and statistics

Supplementary Figure 14. Differentially expressed genes in Dexamethasone versus DMSO control SARC cells

Supplementary Figure 15. Single-cell RNA sequencing of SarBC-01 cells following Dexamethasone treatment

Supplementary Figure 16. Hypothetical model: Glucocorticoid Receptor signaling in SARC tumors

Supplementary Figure 17. Uncropped western blot for main Fig. 3b

Supplementary Tables

Supplementary Data 1. List of somatic mutations (excel file)

Supplementary Data 2. Hight throughput drug screen (excel file)

Supplementary Data 3. IC_{50} and GR_{50} of selected compounds (excel file)

Supplementary Data 4. Patients' cohorts (excel file)

Supplementary Data 5. List of antibodies used in this study (excel file)

Supplementary Videos

Supplementary Video 1. Invasion assay SarBC-01

Supplementary Video 2. Invasion assay UroBC-01

Supplementary Video 3. Invasion assay UroBC-16

Supplementary Video 4. Invasion assay UroBC-22

Supplementary Video 5. Invasion assay SarBC-01 (DMSO treated)

Supplementary Video 6. Invasion assay SarBC-01 (Dexamethasone treated)

Supplementary Figures

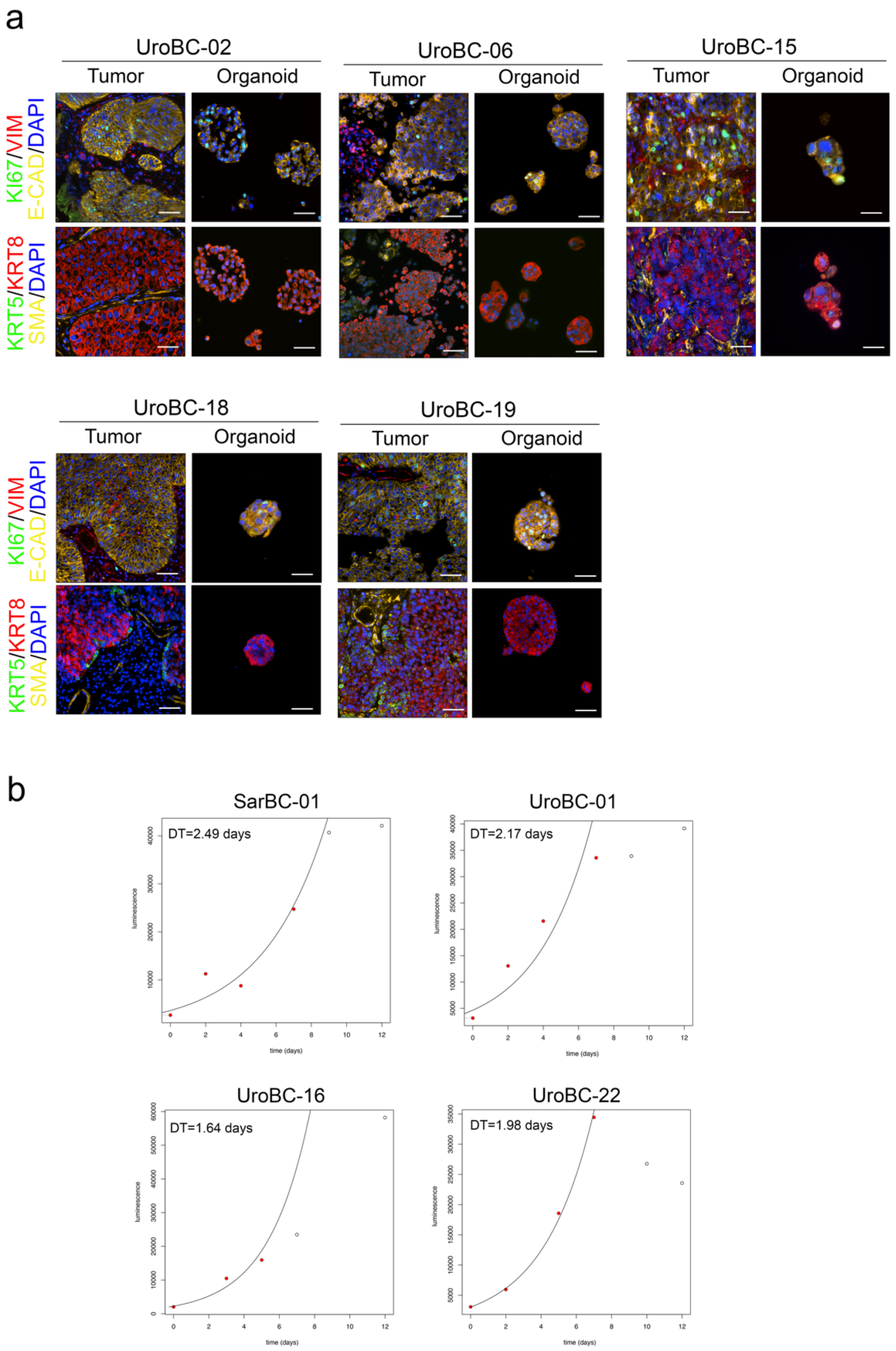

Supplementary Figure 1. Characterization of organoid models. (a) Immunofluorescence analysis of the short-term organoid models UroBC-02, UroBC-06, UroBC-15, UroBC-18, and

UroBC-19 tumor/organoid pairs. Shown are representative images for the indicated antibodies. *Vim: Vimentin, E-cad: E-cadherin, SMA: Smooth Muscle Actin*. DAPI: 4',6-diamidino-2-phenylindole. Scale bars represent 50 μm. **(b)** Dotplots representing the viability (luminescence) of SarBC-01, UroBC-01, UroBC-16, and UroBC22 over the course of 12 days. Red dots represent data points in which cells follow an exponential growth. Exponential curves best fitting viability over time during the exponential growth phase are shown. Doubling times are indicated for each line (DT).

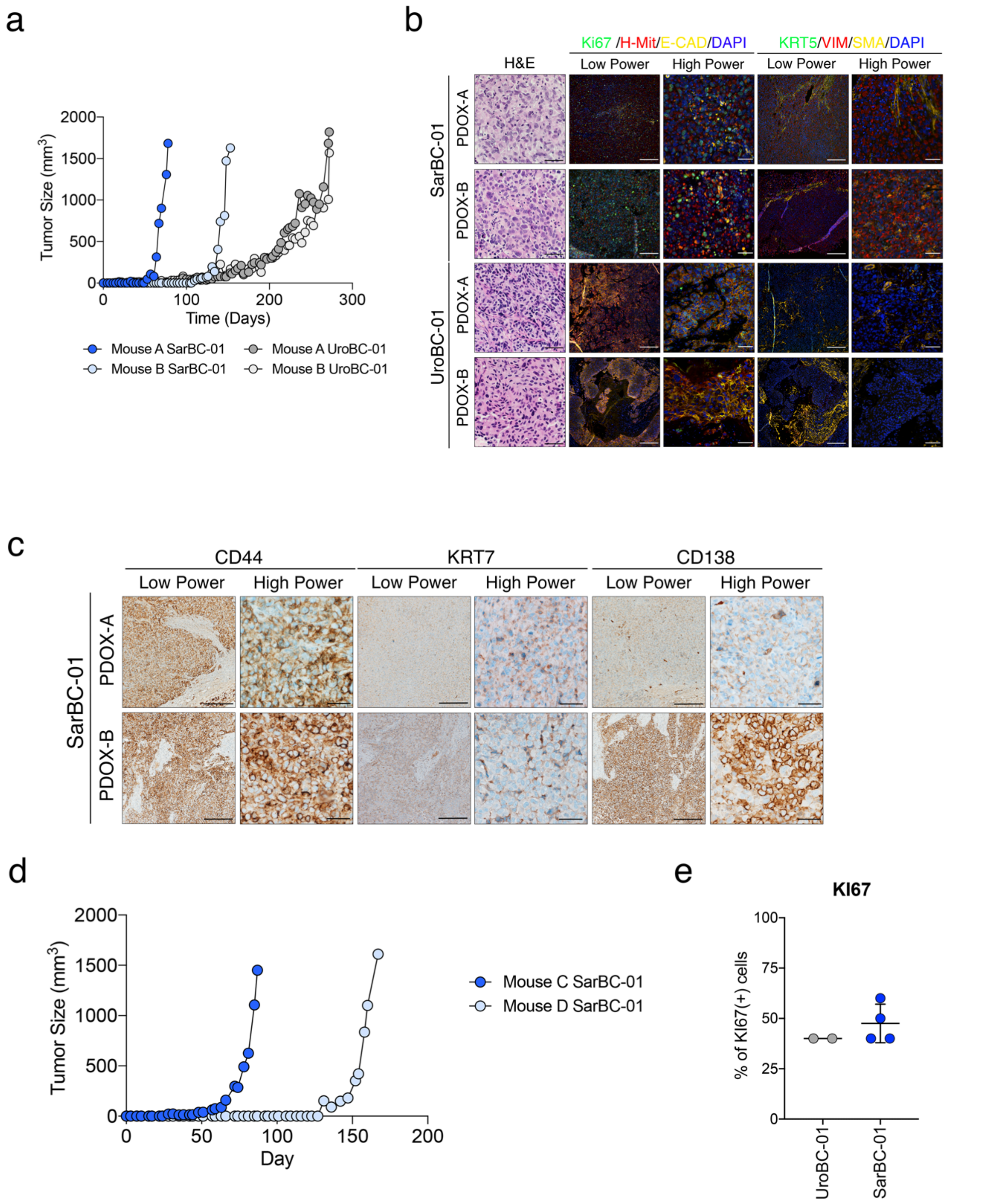

Supplementary Figure 2. Characterization of SarBC-01 and UroBC-01 Patient-Derived Organoid Xenografts (PDOXs). (a) Growth kinetic of xenografts generated by subcutaneous injection of SarcBC-01 and UroBC-01 cells in NSG mice. Two mice were injected per organoid line. **(b)** H&E and immunofluorescence analyses of SarBC-01 (top) and UroBC-01 (bottom) PDOXs. Shown are representative images for the indicated antibodies. *H-Mit: Human mitochondria, Vim: Vimentin, E-cad: E-cadherin, SMA: Smooth Muscle Actin. DAPI: 4',6-*

diamidino-2-phenylindole. Scale bars represent 50 μm for high magnification images and 250 μm for low magnification images. **(c)** Representative IHC staining for the indicated antibodies, showing expression of specific epithelial markers in the xenografts derived from SarBC-01 cells. Scale bars represent 50 μm for high magnification images and 250 μm for low magnification images. **(d)** Growth kinetic of xenografts generated by subcutaneous injection of SarBC-01 cells in two additional NGS mice (PDOX C and D). **(e)** Scatter plot displaying the percentage of $KI67^{+}$ cells in UroBC-01 PDOXs (n=2) and SarBC-01 PDOXs (n=4). Mean and standard deviations are indicated.

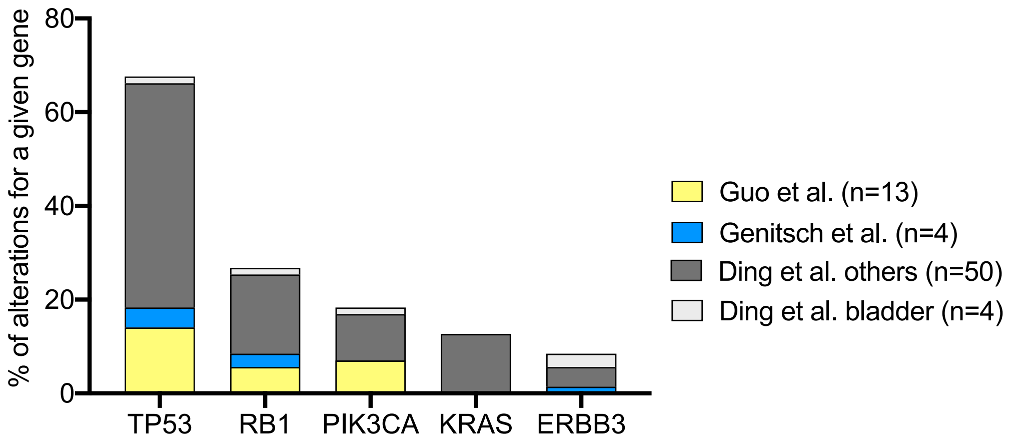

Supplementary Figure 3. Frequency of specific genomic alterations in sarcomatoid tumors. Four datasets were included in the analysis, integrating genomic data obtained from sarcomatoid bladder tumors (*Guo et al., Genitsch et al., Ding et al. bladder*) as well as other sarcomatoid tumor entities (*Ding et al., others*).

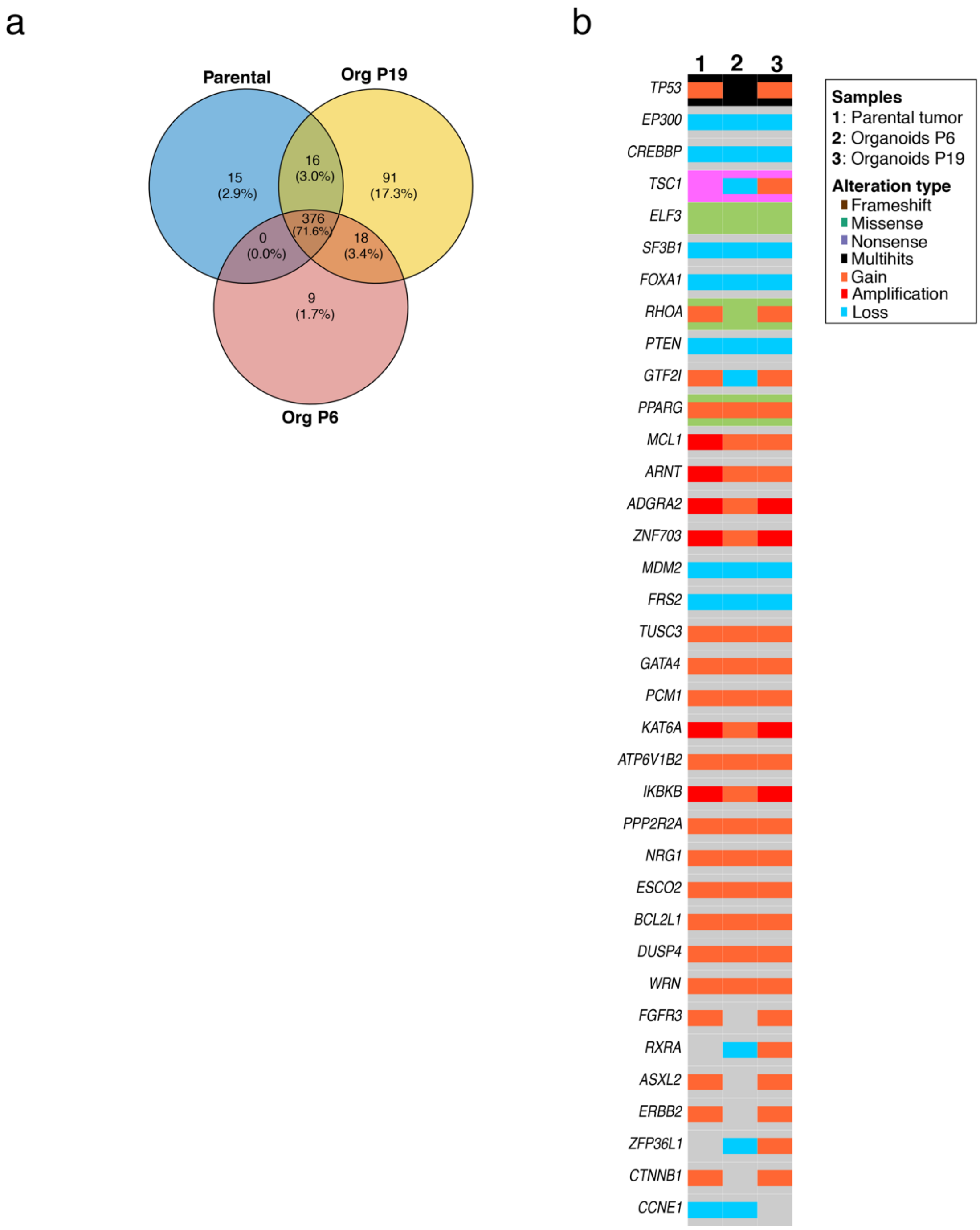

Supplementary Figure 4. Whole Exome Sequencing Analysis of UroBC-01 tumor and matched organoids. (a) Venn diagram depicting the number of shared mutations between UroBC-01 patient tumor and derived organoids at early and late passages (passage 6 and 19, respectively) detected using whole exome sequencing (WES). Numbers in brackets indicate the proportion of shared mutations in each group **(b)** Oncoplots depicting genomic alterations in UroBC-01 tumor and its derived organoids, as revealed by whole exome sequencing. Shown are alterations among the top 100 genes commonly mutated in BC (TCGA). Only

alterations found in at least two samples are represented. A complete list of genomic alterations can be found in **Supplementary Data 1**.

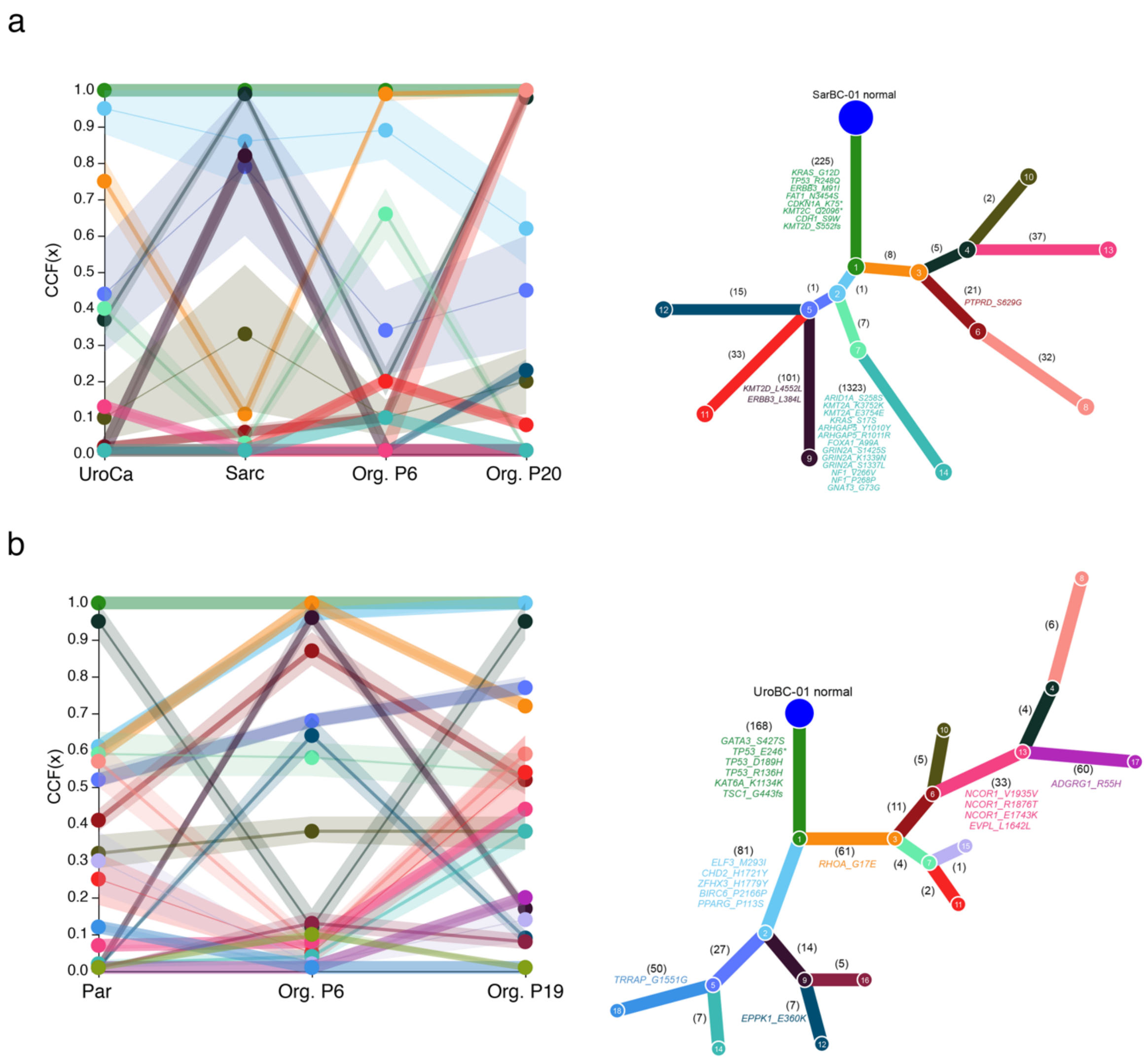

Supplementary Figure 5. Clonal evolution in SarBC-01 and UroBC-01 tumors and derived models. (a) UroCa tumor compartment, SARC tumor compartment, and its derived organoids at passage 6 (Org. P6) and passage 20 (Org. P20) for patient SarBC-01. **(b)** Parental tumor (Par) and its derived organoids at passage 6 (Org. P6) and passage 19 (Org. P19) for patient UroBC-01. For both **(a)** and **(b)**, trace plots (left panels) show cancer cell fraction (CCF) for each mutational cluster in each sample. Ribbons show 95% confidence intervals, while center of bands show mean CCF estimate. Phylogenetic trees (right panels) show best solution for evolutionary relationship between clones with different clusters of mutations where each node (numbered) is a cluster of mutations. Numbers on each branch show the number of mutations distinguishing a clone from the previous clone (all genes). Potential driver genes mutations distinguishing between a clone and the previous are indicated in colors corresponding to the branch.

a

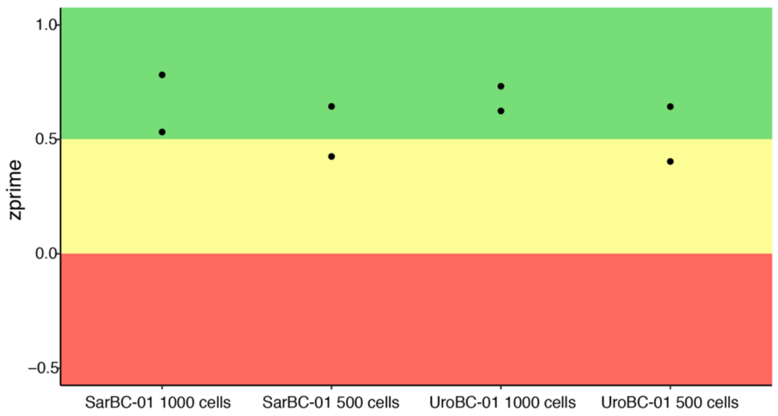

b

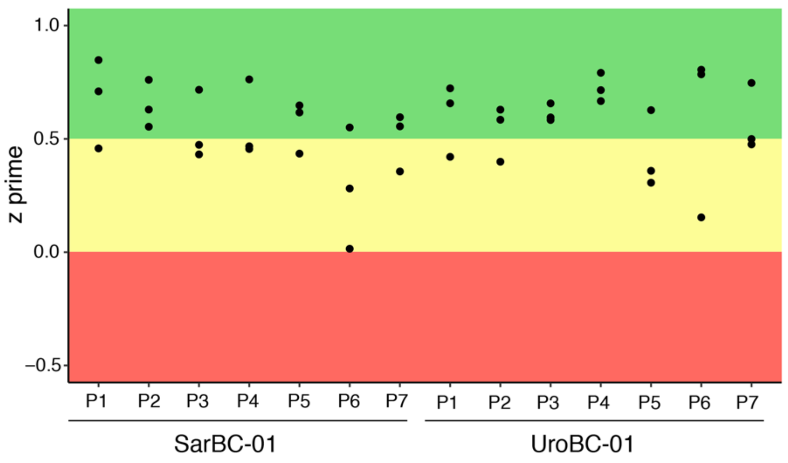

Supplementary Figure 6. Optimization and quality control of the high-throughput drug screen. (a) For optimization of the screen, densities of 500 cells and 1000 cells per well were tested. Z prime values were calculated for each line for both densities. Assays with Z prime values above 0.5 are considered as excellent quality (green area), while assays with Z prime values between 0 and 0.5 are considered as good quality (yellow area). **(b)** For quality control of the screen, Z prime values were calculated based on 14 negative and positive controls for each plate (P1-P7, <0.01 % DMSO) for each line.

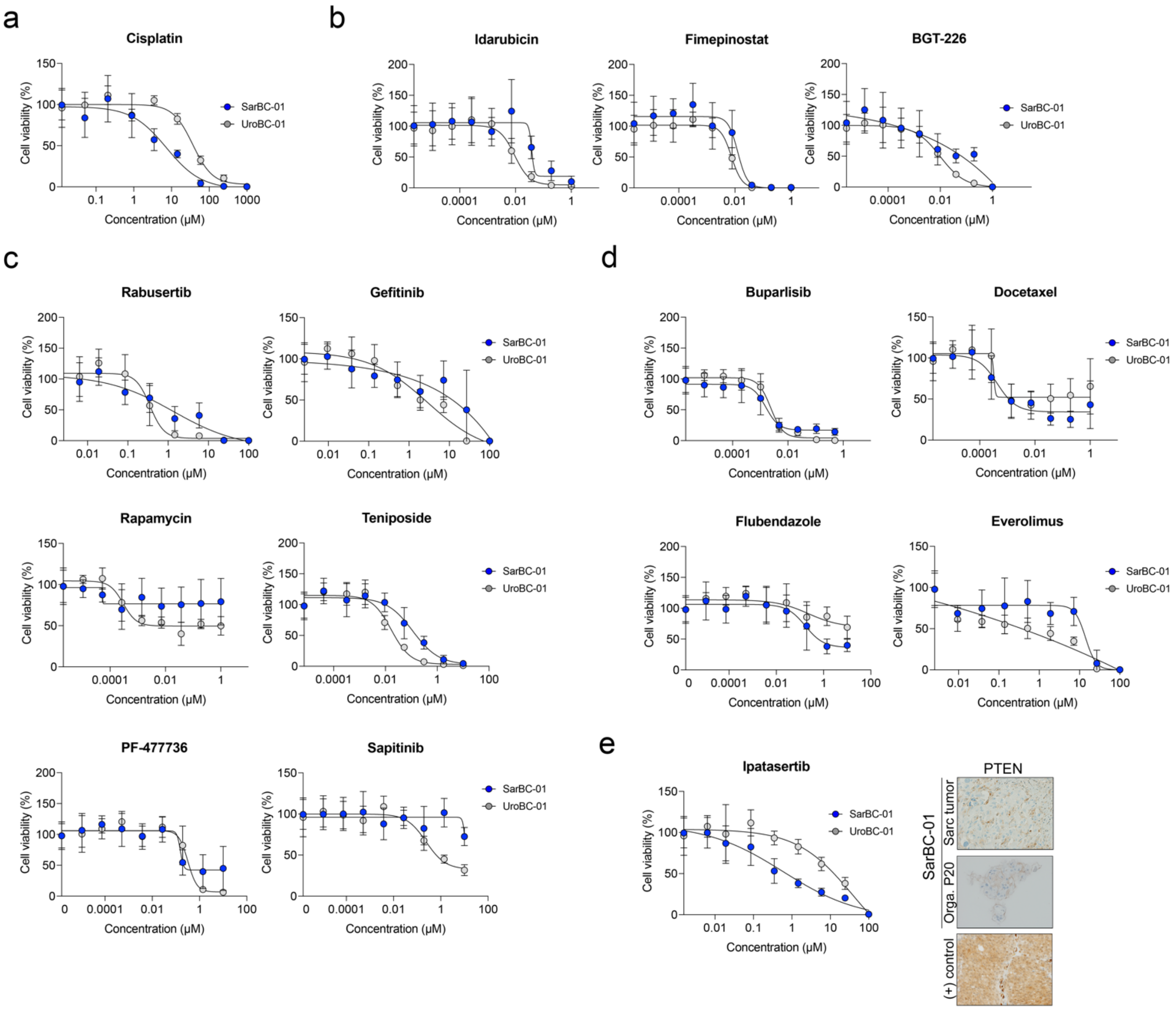

Supplementary Figure 7. Drug response analysis for additional selected compounds in SarBC-01 and UroBC-01. Dose responses curves for SarBC-01 (blue dots) and UroBC-01 (grey dots) organoids treated with a selected panel of drugs. Examples include **(a)** cisplatin, a standard of care compound that was not identified as a “hit” in the drug screen, **(b)** compounds identified as “hits” in both UroBC-01 and SarBC-01, **(c)** compounds identified as “hits” in UroBC-01 only, **(d)** compounds identified as “hits” in SarBC-01 only. **(e)** SarBC-01 cells are responsive to the AKT inhibitor ipatasertib (**left panel**), which may be explained by a loss of PTEN expression at protein level (**right panel**). Positive control for intact PTEN expression is

also shown. IC_{50} and GR_{50} values for tested compounds are reported in **Supplementary Data 3**, whenever available.

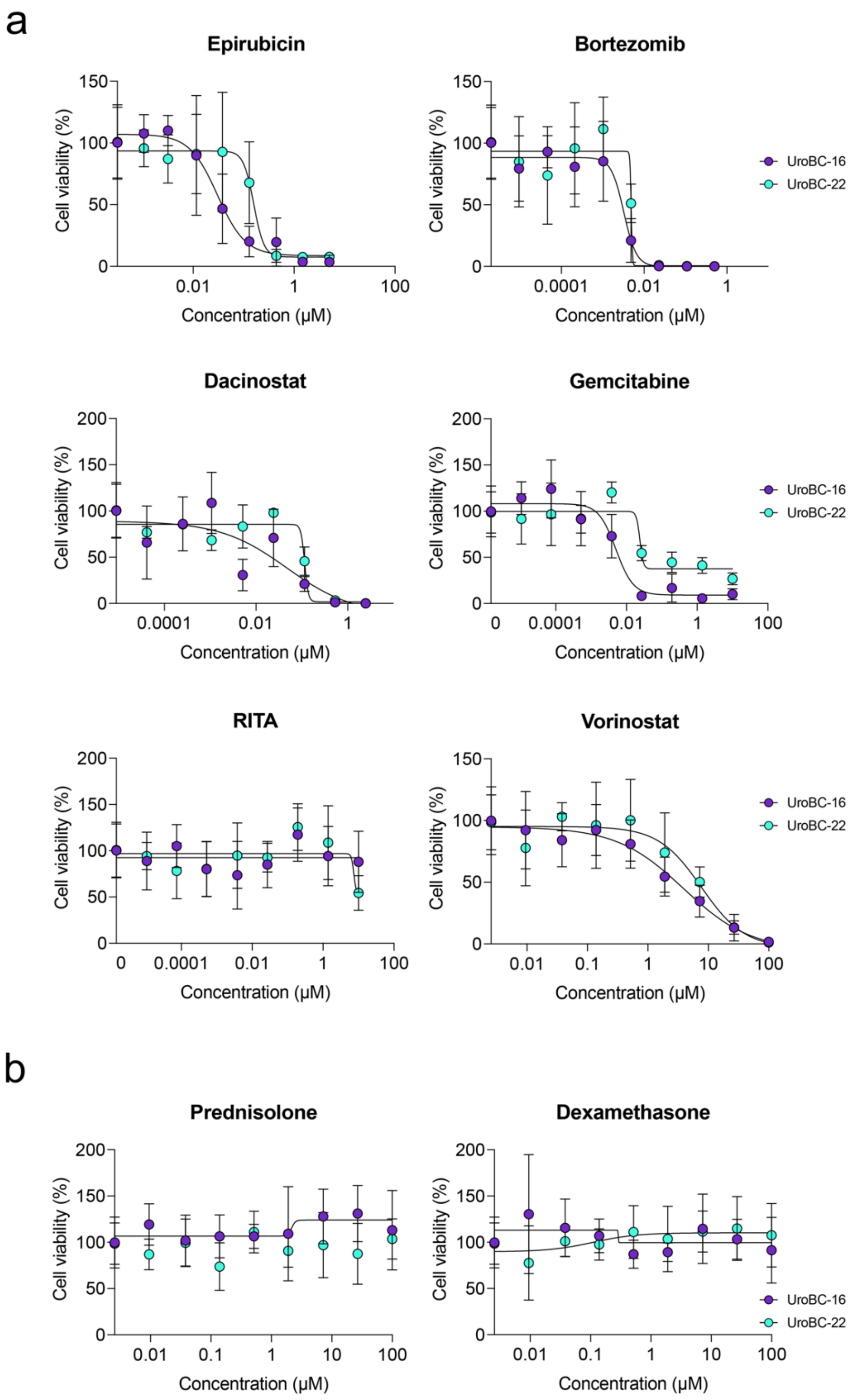

Supplementary Figure 8. Drug response analysis for selected compounds in UroBC-16 and UroBC-22. Dose responses curves for two additional UroCa-derived PDO models: UroBC-16 (purple dots) and UroBC-22 (cyan dots). **(a)** The selected panel of drugs includes

compounds identified as “hit” in both SarBC-01 and UroBC-01 lines (epirubicin, bortezomib, dacinostat), in UroBC-01 only (gemcitabine), or in SarBC-01 only (RITA, vorinostat). IC_{50} values for all tested compounds are reported in **Supplementary Data 3,** whenever available. **(b)** Prednisolone and dexamethasone have no significant effects on the viability of UroBC-16 and UroBC-22 organoids.

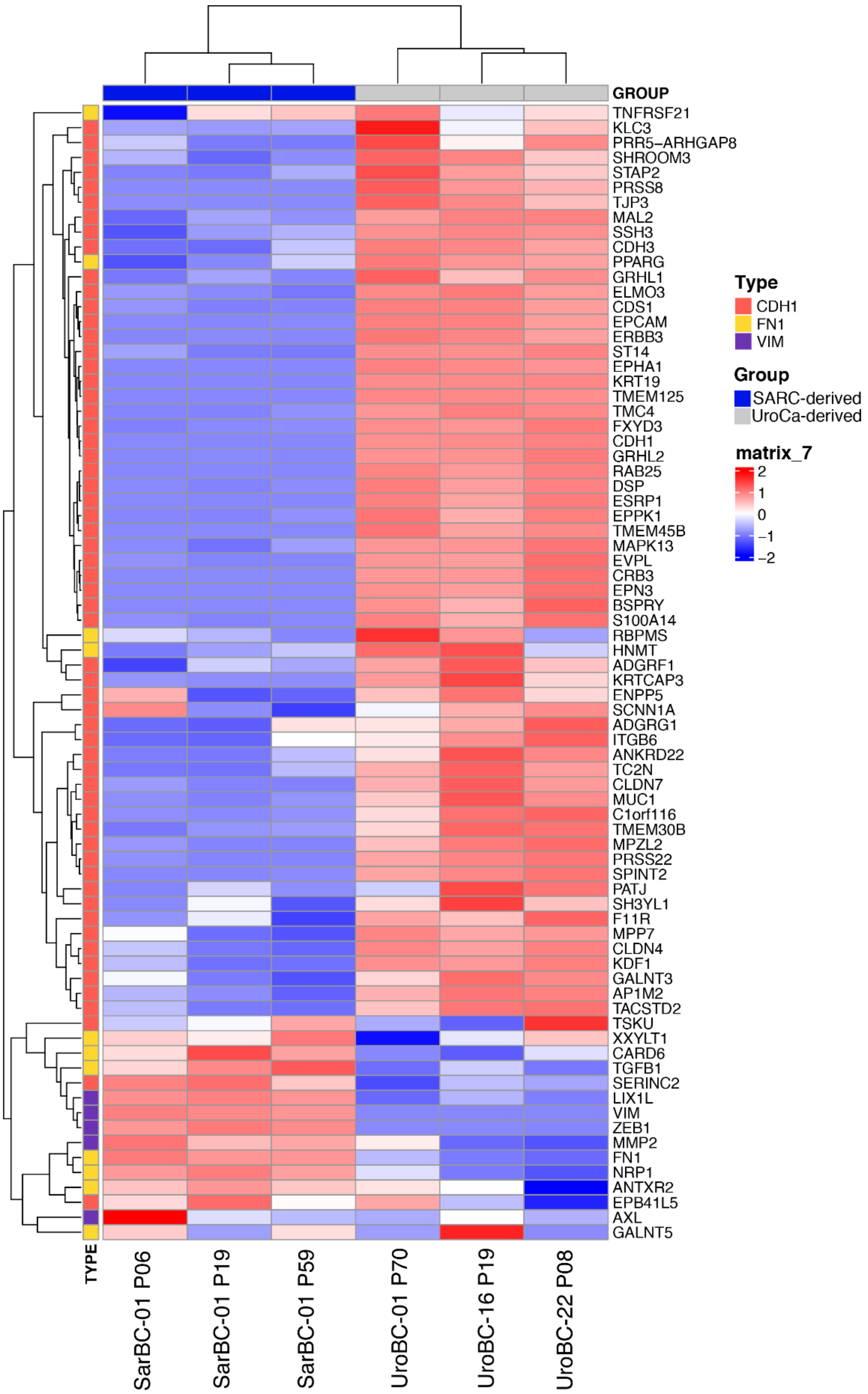

Supplementary Figure 9. Expression of EMT-associated genes in SARC vs. UroCa organoids. Heatmap displaying unsupervised clustering and normalized gene expression

level (Z-score) of SarBC-01 organoids (n=3) and UroCa-derived PDO models (n=3). mRNA from SarBC-01 organoids was extracted at different passages (passage 6, passage 19, passage 59). Selected genes belong to a signature of epithelial-to-mesenchymal transition and correlate with the expression of VIM, CDH1 or FN1 (*Byers et al., 2013*)

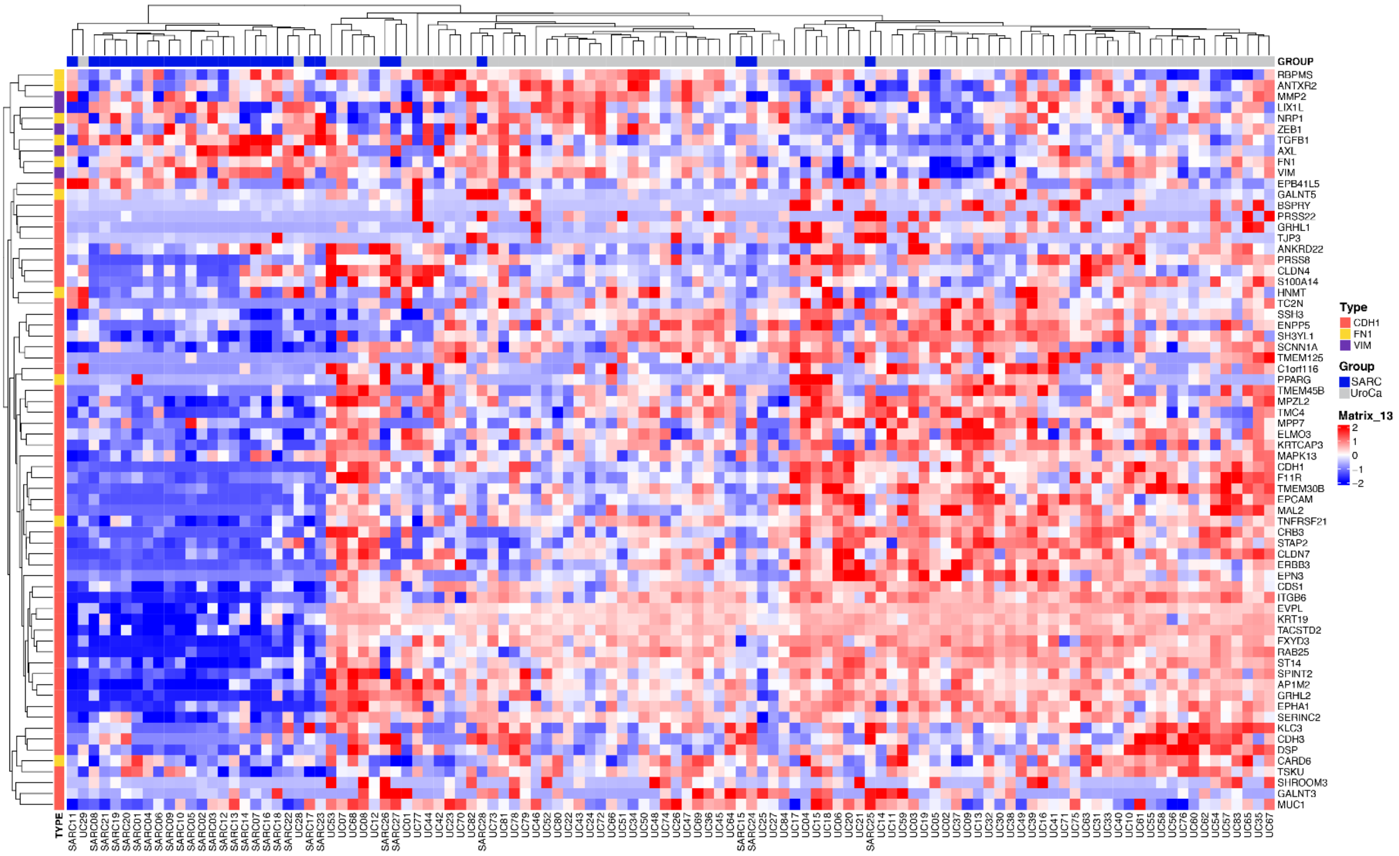

Supplementary Figure 10. Expression of EMT-associated genes in SARC vs. UroCa tumors. Heatmap displaying unsupervised clustering and normalized gene expression level (Z-score) of SARC primary tumors (n=28) and UroCa primary tumors (n=84) obtained from *Guo et al., 2019*. Selected genes belong to a signature of epithelial-to-mesenchymal transition and correlate with the expression of VIM, CDH1 or FN1 (*Byers et al., 2013*)

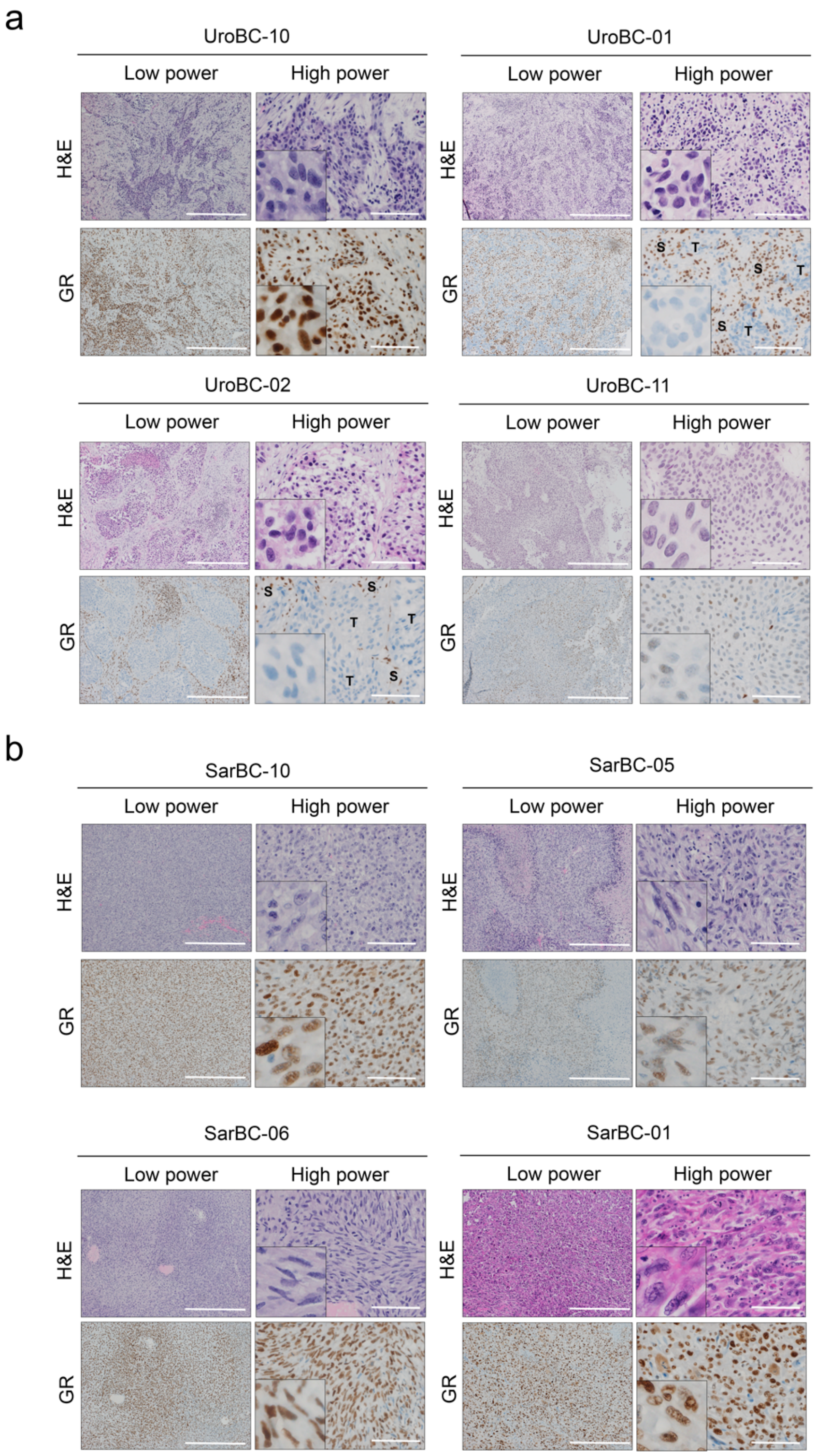

Supplementary Figure 11. Glucocorticoid Receptor (GR) expression in UroCa and SARC samples. H&E and immunohistochemical staining for GR in UroCa samples **(a)** and SARC samples **(b).** *S: Stroma, T: tumor.* Scale bars represent 100 μm for high magnification images and 500 μm for low magnification images.

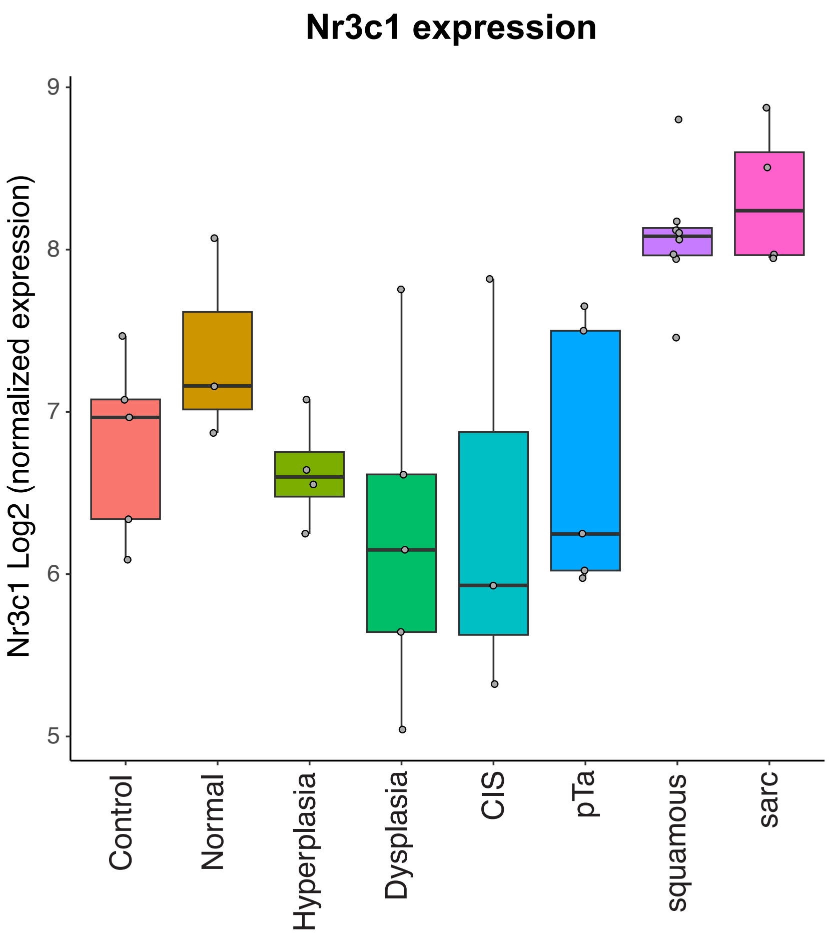

Supplementary Figure 12. Expression of *nr3c1* in a mouse model of bladder cancer progression. Data were obtained from a dataset downloaded from *Fontugne et al., 2023.* Shown are samples from mouse controls (i.e. not BBN treated, n=5), normal (BBN treated with normal histology, n=3), hyperplasia (n=4), dysplasia (n=4), carcinoma-in-situ (CIS, n=3), pTa (low-grade UroCa, n=5), basal/squamous (n=8), and sarcomatoid BC (sarc, n=4).

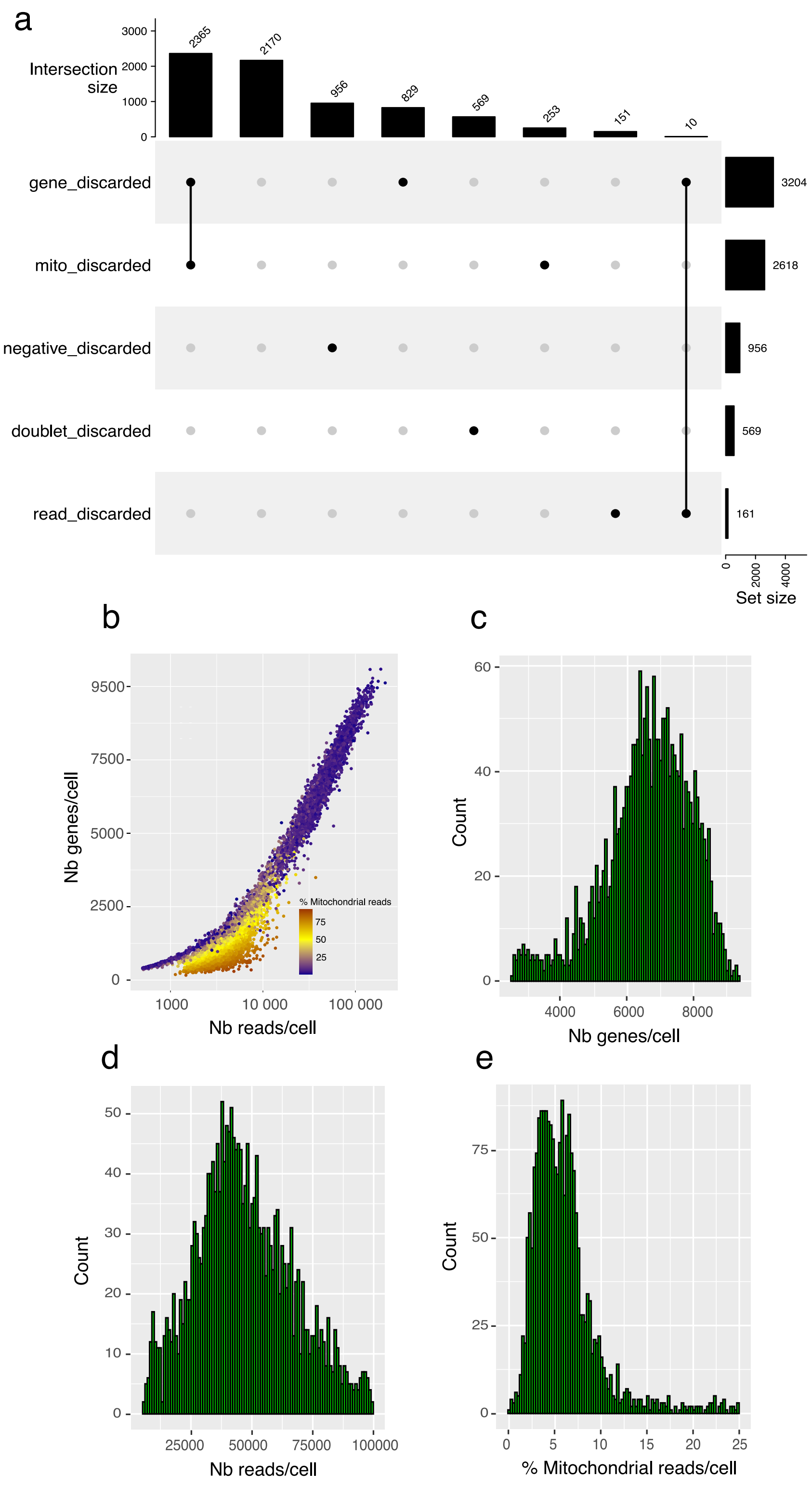

Supplementary Figure 13. scRNA-seq quality controls and statistics. (a) UpSet plot showing numbers of discarded cells based on different quality filters (*i.e* number of genes, number of reads and percentage of mitochondrial reads per cell), and demultiplexing filters related to the MULTI-seq strategy (*i.e* doublets and negatives). Cells that passed all quality

filters and were further analysed are in the second column of the graph (n = 2170). **(b)** Scatter plot displaying the relationship between the number of reads (*i.e* library size) per cell on the x-axis and the number of detected genes per cell on the y-axis. The dots are color-coded based on the percentage of mitochondrial reads observed in each cell. Histograms illustrating the distributions of either **(c)** the number of genes detected per cell, or **(d)** the number of reads per cell, or **(e)** the percentage of mitochondrial reads per cell, observed after applying all quality and demultiplexing filters**.**

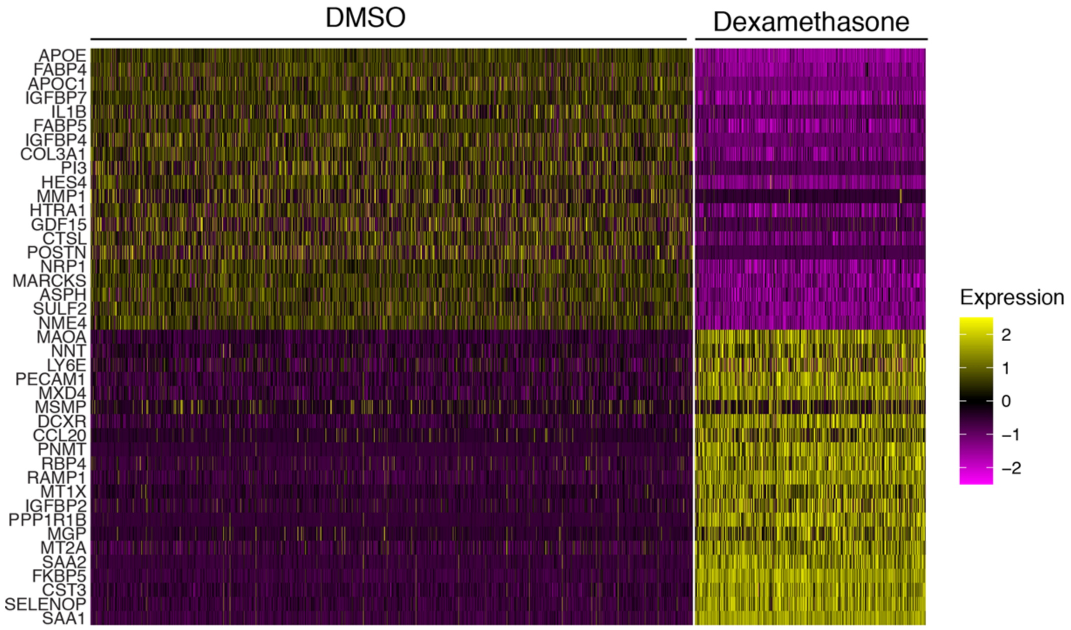

Supplementary Figure 14. Differentially expressed genes in Dexamethasone- versus DMSO-treated SarBC-01 cells. Heatmap displaying top 20 up- and down-regulated genes in dexamethasone- vs DMSO-treated SarBC-01 spheroids without random down sampling of DMSO-treated condition.

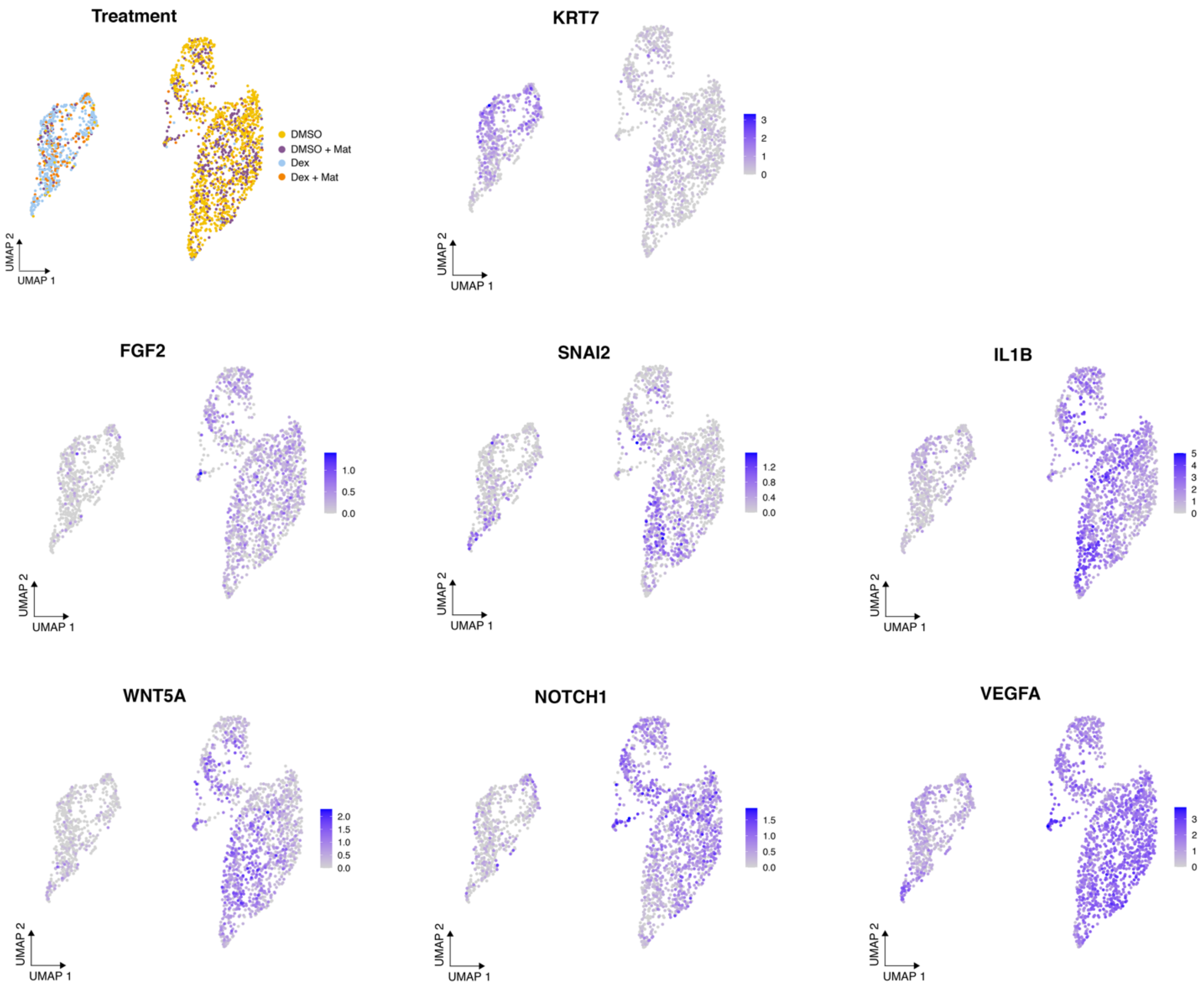

Supplementary Figure 15. Single-cell RNA sequencing of SarBC-01 cells following dexamethasone treatment. Shown are UMAP representations of the treatment conditions and expression of selected indicated genes. 2170 cells were analyzed following incubation with DMSO or dexamethasone (Dex) in invasion assay conditions and in presence of absence of Matrigel (Mat), leading to 4 different culture conditions (UMAP "treatment": DMSO, DMSO+Mat, Dex, Dex+Mat; see Material and Methods for more details).

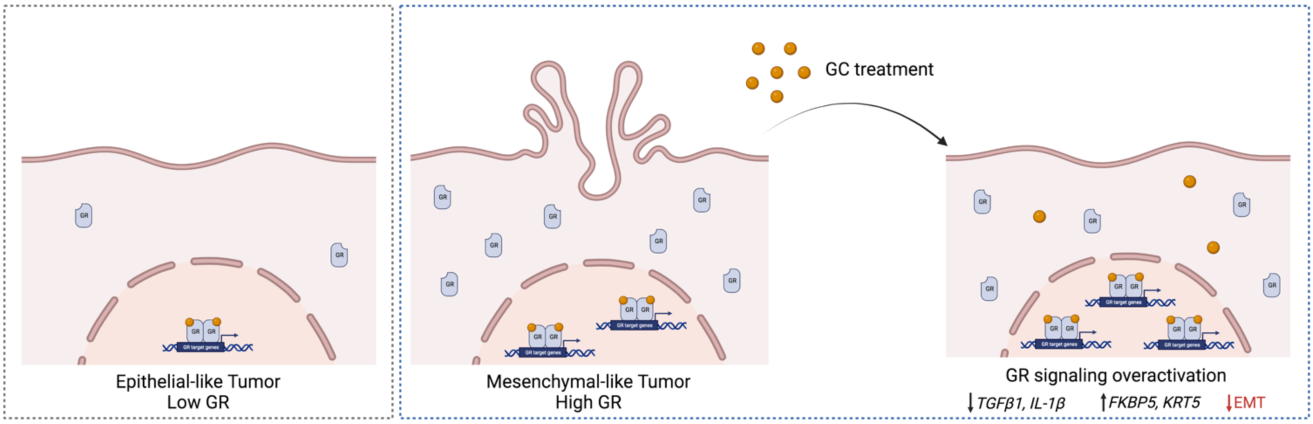

Supplementary Figure 16. Hypothetical model: Glucocorticoid Receptor signaling in SARC tumors. Mesenchymal-like bladder tumors such as SARC, which display increased glucocorticoid receptor (GR) expression, may be more sensitive to glucocorticoid (GC) treatment. Treatment with GC leads to overactivation of the GR signaling pathway, as observed by *FKBP5* increase; this is accompanied by a reduction of expression of genes associated with EMT (e.g. *TGFB1*), an increase expression of epithelial-associated genes (e.g. *KRT5, KRT7*), and the acquisition of epithelial-like morphological features. *Created with BioRender.com.*

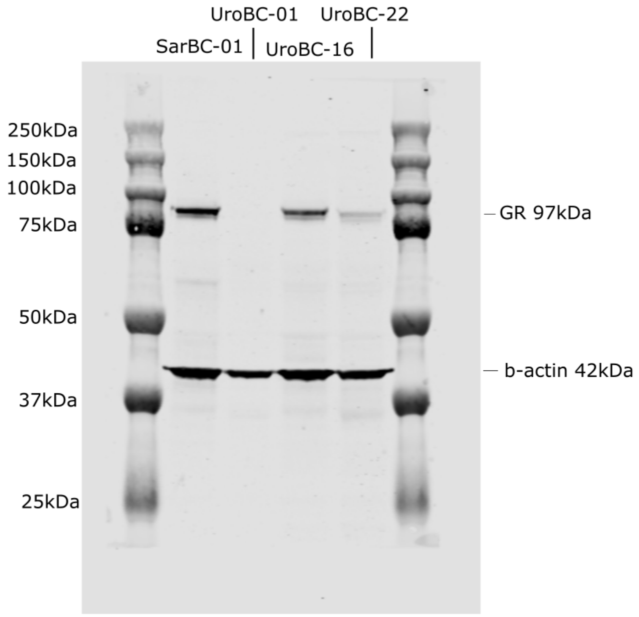

Supplementary Figure 17. Uncropped western blot for main Fig. 3b
