# Supplementary material for: Patient-derived organoids identify tailored therapeutic options and determinants of plasticity in sarcomatoid urothelial bladder cancer
Source: NPJ Precis Oncol. 2023 Nov 2;7:112. doi: 10.1038/s41698-023-00466-w (PMC10622543; doi:10.1038/s41698-023-00466-w)
Supplement: Supplementary file 13 — REPORTING SUMMARY [file 41698_2023_466_MOESM13_ESM.pdf]

nature portfolio

Corresponding author(s): Clementine LE MAGNEN

Last updated by author(s): Aug 15, 2023

Reporting Summary

Nature Portfolio wishes to improve the reproducibility of the work that we publish. This form provides structure for consistency and transparency in reporting. For further information on Nature Portfolio policies, see our Editorial Policies and the Editorial Policy Checklist.

Statistics

For all statistical analyses, confirm that the following items are present in the figure legend, table legend, main text, or Methods section.

n/a	Confirmed	
☐	☒	The exact sample size (*n*) for each experimental group/condition, given as a discrete number and unit of measurement
☐	☒	A statement on whether measurements were taken from distinct samples or whether the same sample was measured repeatedly
☐	☒	The statistical test(s) used AND whether they are one- or two-sided<br>*Only common tests should be described solely by name; describe more complex techniques in the Methods section.*
☒	☐	A description of all covariates tested
☒	☐	A description of any assumptions or corrections, such as tests of normality and adjustment for multiple comparisons
☐	☒	A full description of the statistical parameters including central tendency (e.g. means) or other basic estimates (e.g. regression coefficient) AND variation (e.g. standard deviation) or associated estimates of uncertainty (e.g. confidence intervals)
☒	☐	For null hypothesis testing, the test statistic (e.g. *F*, *t*, *r*) with confidence intervals, effect sizes, degrees of freedom and *P* value noted<br>*Give P values as exact values whenever suitable.*
☒	☐	For Bayesian analysis, information on the choice of priors and Markov chain Monte Carlo settings
☒	☐	For hierarchical and complex designs, identification of the appropriate level for tests and full reporting of outcomes
☒	☐	Estimates of effect sizes (e.g. Cohen's *d*, Pearson's *r*), indicating how they were calculated

Our web collection on statistics for biologists contains articles on many of the points above.

Software and code

Policy information about availability of computer code

Data collection

Immunofluorescence images were captured on a Nikon Ti2 microscope using NIS-Element software (Nikon).
Immunohistochemistry images were captured using CellSens Dimension software (Olympus).
Invasion assay was quantified using Incucyte® Live-Cell Analysis System (Sartorius).
Western Blot membranes were scanned using the Odyssey Infrared Imaging System (LI-COR Biosciences).
Luminescence readouts were acquired using a Synergy H1 Multi-Mode Reader (BioTek Instruments).
Sequencing data were generated on a Illumina NovaSeq 6000 platform.

Data analysis	Pre-processing and variants annotation of whole exome sequencing data was performed using bwa v.0.7.12, Mutect2 and SnpEff v.4.1. Clonal analysis was performed using FACETS v.0.5.6 and ABSOLUTE v.2.0.<br><br>Pre-processing of bulk RNA sequencing data was performed using STAR v.2.7.9a, RSEM v.1.3.3.<br><br>Pre-processing of single-cell RNA sequencing was performed using Cellranger v.6.1.2.<br><br>Further data analyses were performed using R v.4.1.2. R package 'growth rate' v.0.8.4 was used to assess cell proliferation. Heatmaps for whole exome sequencing were generated using R package 'maftools' v.2.10.5. Differential gene expression analysis was performed using R package 'DeSeq' v.3.17 and heatmaps were generated using R package 'pheatmap' v.1.0.12. Single-cell RNA sequencing data were analyzed using R package 'Seurat' v.4.3.0.<br><br>Prism GraphPad software v.8.0 was used for remaining statistical analyses and data representation.

For manuscripts utilizing custom algorithms or software that are central to the research but not yet described in published literature, software must be made available to editors and reviewers. We strongly encourage code deposition in a community repository (e.g. GitHub). See the Nature Portfolio guidelines for submitting code & software for further information.

Data

Policy information about availability of data

All manuscripts must include a data availability statement. This statement should provide the following information, where applicable:

- Accession codes, unique identifiers, or web links for publicly available datasets
- A description of any restrictions on data availability
- For clinical datasets or third party data, please ensure that the statement adheres to our policy

The whole exome sequencing and the single-cell RNA sequencing data generated in this paper have been deposited to the European Genome-phenome Archive (Accession number EGAS000011007430). The use of the data will be subjected to agreement of a data use policz which details the minimum protecion measures required to data encrzption and user access.

Research involving human participants, their data, or biological material

Policy information about studies with human participants or human data. See also policy information about sex, gender (identity/presentation), and sexual orientation and race, ethnicity and racism.

Reporting on sex and gender	For phenotypical and functional studies, we reported the use of 8 organoids models generated from 8 patients including 7 males and 1 female. For glucocorticoid receptor IHC analysis, we used an archival cohort of FFPE samples derived from 27 patients including 18 males and 9 females. Sex and gender were not included in the experimental design and were not used as covariates for statystical analysis.
Reporting on race, ethnicity, or other socially relevant groupings	We did not report data regarding race, ethnicity, or other socially relevant groupings for our cohorts as these categories were not included in the experimental design and were not used as covariates for statistical analysis.
Population characteristics	Details for the cohort of 8 patients are presented in a table in material and methods. Details for the cohort of 27 patients are presented in supplementary table 5.
Recruitment	Recruitment was performed after collection of informed consent among bladder cancer patients undergoing transurethral resection of bladder tumor or radical cystectomy at the University Hospital of Basel. Single-center recruitment may have introduced a bias in the population composition.
Ethics oversight	8 patient cohort - Ethical Committee of Northwestern and Central Switzerland (EKBB 37/13).<br>27 patient cohort - Ethical Committee of Northwestern and Central Switzerland (EKNZ 2014-313).

Note that full information on the approval of the study protocol must also be provided in the manuscript.

Field-specific reporting

Please select the one below that is the best fit for your research. If you are not sure, read the appropriate sections before making your selection.

☒ Life sciences ☐ Behavioural & social sciences ☐ Ecological, evolutionary & environmental sciences

For a reference copy of the document with all sections, see nature.com/documents/nr-reporting-summary-flat.pdf

Life sciences study design

All studies must disclose on these points even when the disclosure is negative.

Sample size	No sample-size calculations were performed. The sample size was dependent on the availability of material for our study. We included phenotypical analysis for 8 organoid lines and functional analysis for a subset of 4 organoid lines that we were able to maintain and expand in

	culture for more than one month. For GR quantification we used an archival cohort of 27 FFPE bladder cancer samples, 14 displaying epithelial morphology and 13 displaying a sarcomatoid phenotype.
Data exclusions	No data were excluded from the study.
Replication	Every model was considered as a single biological replicate. All in vitro experiments were conducted at least twice, excluding RNA and DNA sequencing (single experiment). Experiment replications were succesful. In drug screens and dose-response experiments, each drug condition was tested in at least 3 technical replicates.
Randomization	No randomization was performed for this study. Phenotypical analyses were direct comparisons of single organoid models. Drug screen and dose-response analyses were optimized to avoid possible confounders such as edge effects.
Blinding	IHC quantification of GR in the archival cohort was performed in single-blind. No blinding was applicable for the comparison analysis among the single organoid models.

Reporting for specific materials, systems and methods

We require information from authors about some types of materials, experimental systems and methods used in many studies. Here, indicate whether each material, system or method listed is relevant to your study. If you are not sure if a list item applies to your research, read the appropriate section before selecting a response.

Materials & experimental systems

n/a	Involved in the study
☐	☒ Antibodies
☒	☐ Eukaryotic cell lines
☒	☐ Palaeontology and archaeology
☐	☒ Animals and other organisms
☒	☐ Clinical data
☒	☐ Dual use research of concern
☒	☐ Plants

Methods

n/a	Involved in the study
☒	☐ ChIP-seq
☒	☐ Flow cytometry
☒	☐ MRI-based neuroimaging

Antibodies

Antibodies used	A complete list of antibodies and concentrations used in this study is provided in Supplementary Table 5.
Validation	IHC Antibodies:<br>immunohistochemistry staining were performed with antibodies validated for diagnostic purposes at University Hospital of Basel.<br><br>IF Antibodies:<br>Cytokeratin 5: Yuspa et al. (doi 10.1083/jcb.109.3.1207)<br>Cytokeratin 8: Memarzadeh et al. (doi 10.1073/pnas.1012548107)<br>α-SMA: Shirai et al. (doi 10.1038/s41598-022-24957-1)<br>KI67: Zhang et al. (doi 10.4103/1673-5374.314322)<br>E-Cadherin: Namiki et al. (doi 10.1038/s41598-023-27859-y)<br>Glucorticoid Receptor: Tertil et al. (doi 10.1038/s41398-018-0300-x)<br>Vimentin: Yao et al. (doi 10.1016/j.exer.2021.108864)<br>Human Mitochondria: Alexander et al. (doi 10.1002/adhm.202102153)

Animals and other research organisms

Policy information about studies involving animals; ARRIVE guidelines recommended for reporting animal research, and Sex and Gender in Research

Laboratory animals	NOD-scid-gc (NSG) mice, in average 6 weeks old.
Wild animals	This study did not involve wild animals.
Reporting on sex	The mice used for this study were all males. Sex was not included in the experimental design and was not used as covariate for statystical analysis.
Field-collected samples	This study did not involve field-collected samples.
Ethics oversight	Animal Care Committee of the Kanton Basel-Stadt, Switzerland (3066-32428).

Note that full information on the approval of the study protocol must also be provided in the manuscript.
